# Prediction of disability-adjusted life years for diseases due to low fruit intake in 2017–2040 in Japan

**DOI:** 10.1017/S1368980020004541

**Published:** 2021-07

**Authors:** Daisuke Yoneoka, Shuhei Nomura, Shiori Tanaka, Aya Ishizuka, Ueda Peter, Santosh Kumar Rauniyar, Keiji Nakamura, Hisayuki Uneyama, Naoki Hayashi, Kenji Shibuya

**Affiliations:** 1 Graduate School of Public Health, St. Luke’s International University, Tokyo, Japan; 2 Department of Global Health Policy, Graduate School of Medicine, The University of Tokyo, Tokyo, Japan; 3 Department of Health Policy and Management, School of Medicine, Keio University, 35 Shinanomachi, Shinjuku-ku, Tokyo 160-8582, Japan; 4 Epidemiology and Prevention Group, Center for Public Health Sciences, National Cancer Center, Tokyo, Japan; 5 Department of Medicine, Clinical Epidemiology Division, Karolinska Institutet, Stockholm, Sweden; 6 Graduate School of Environmental and Information Studies, Tokyo City University, Yokohama, Japan; 7 Ajinomoto Co., Inc., Tokyo, Japan; 8 Department of Applied Biological Chemistry, Graduate School of Agriculture and Life Sciences, The University of Tokyo, Tokyo, Japan; 9 Department of Global Health Policy, Institute for Population Health, King’s College London, London, UK

**Keywords:** DALY rate, Low fruit intake, Neoplasms, Cardiovascular diseases, Diabetes, Kidney diseases, Japan

## Abstract

**Objective:**

The current study aimed to predict disability-adjusted life years (DALY) rate in Japan through 2040 with plausible future scenarios of fruit intake for neoplasms, cardiovascular diseases (CVD) and diabetes and kidney diseases (DKD).

**Design:**

Data from National Health and Nutrition Surveys and the Global Burden of Diseases study in 2017 were used. We developed an autoregressive integrated moving average model with four future scenarios. Reference scenario maintains the current trend. Best scenario assumes that the goal defined in Health Japan 21 is achieved in 2023 and is kept constant afterwards. Moderate scenario assumes that the goal is achieved in 2040. Constant scenario applies the same proportion of 2016 for the period between 2017 and 2040.

**Setting:**

DALY rates in Japan were predicted for the period between 2017 and 2040.

**Participants:**

Population aged more than than 20 years old.

**Results:**

In our reference forecast, the DALY rates in all-ages group were projected to be stable for CVD and continue increasing for neoplasms and DKD. Age group-specific DALY rates for these three disease groups were forecasted to decrease, with some exceptions. Among men aged 20–49 years, DALY attributable to CVD differed substantially between the scenarios, implying that there is a significant potential for reducing the burden of CVD by increasing fruit intake at the population level.

**Conclusions:**

Our scenario analysis shows that higher fruit intake is associated with lower disease burden in Japan. Further research is required to assess which policies and interventions can be used to achieve an increase in fruit intake as modelled in the scenarios of the current study.

Global Burden of Disease Study (2017) has shown significant associations between low fruit intake and the risk of neoplasms, cardiovascular diseases and diabetes and kidney diseases^(1)^. The Global Burden of Disease analysis in Japan assessed the impact of sixty-seven risk factors, including behavioural, metabolic, environmental and occupational factors, on disease burden measured as disability-adjusted life years (DALY). One DALY can be considered as one lost year of ‘healthy’ life. The study found that low fruit intake was the ninth most important risk factor^(1,2)^ accounting for 2·3 % of the total disease burden^(1)^. In the current study, we take the same definition for fruits as the Global Burden of Disease study, where ‘fruit’ means fresh, frozen, cooked, canned or dried fruits, excluding fruit juices and salted or pickled fruits.

People in Japan eat less fruits as compared with other national populations^(3)^. According to the Food and Agriculture Organisation (FAO) of the UN, in 2013, the average fruit supply per capita in Japan was 144·8 g/d, corresponding to a 135th place among 175 countries. Accordingly, the Japanese fruit supply per person was approximately half of the amount in the USA^(3)^. Japan’s National Health and Nutrition Survey showed that the average Japanese fruit intake per capita peaked at 193·5 g/d in 1975^(4)^. Since then, the intake has been declining and, for the first time in the past 40 years, has dropped below 100 g to reach 98·9 g in 2016^(5)^. In Japan, fruit intake increased with age, and women consumed more fruit than men in all 10-year-old age groups in 2015^(6)^. The national health promotion policy guideline ‘Health Japan 21 (second phase)’, which was established in 2012 to improve lifestyle and extend healthy life expectancy, has set a target of having <30 % of the population consuming <100 g of fruit/d^(7)^. However, even among those who consumed the most fruit, identified as those in their 70s, only 32 % met the recommended level^(6)^. This target, defined on the population level, is not comparable with the individual-level goal defined in the World Health Organisation (WHO)’s guideline that recommends eating at least 400 g of fruit and vegetables per day (excluding potatoes and other starchy tubers)^(8)^.

Based on four different scenarios for future fruit consumption in Japan, the current study aims to predict the DALY through 2040 (when many Japanese health policies, such as ‘Social Security and Workplace Reform with a View to 2040’^(9)^ and ‘Healthy Life Extension Plan’^(10)^, often set their goal to be achieved) for chronic diseases that are well known to be associated with low fruit intake^(1)^. Since low fruit intake is a modifiable risk factor, medical and political interventions to modify this risk factor could have a large potential to prevent disease, prolong healthy life and efficiently maintain and improve population health^(11)^. By providing estimates of the effects of altered fruit consumption, the current study aims to provide data that could guide decisions on policy design and prioritisation.

## Methods

### Overview

According to the Global Burden of Disease study, the three disease groups, or three leading causes of health lost, that have been identified to be associated with low fruit intake were neoplasms, cardiovascular diseases and diabetes and kidney diseases from level 2 in the Global Burden of Disease hierarchical causal structure. The data for these diseases from 1990 to 2016 were used to predict the future values of DALY rates for the period from 2017 to 2040.

Following the Global Burden of Disease’s prediction methodology^(12)^, we developed a three-component model of cause-specific DALY for these three causes. This model included a component explained by changes in major behavioural and metabolic risk predictors including the proportion of those who consume less than 100 g of fruit; a component explained by income per person, educational attainment and total fertility rate under 25 years, which were aggregated into the socio-demographic index that ranges from 0 to 1 and an autoregressive integrated moving average model to estimate the unexplained component over the time span. Further details, including data sources and model formulae, are explained below.

### Data sources

#### Disability-adjusted life years and socio-demographic index data, 1990–2016

The estimates of the DALY rate (per 100 000 population) for neoplasms, cardiovascular diseases and diabetes and kidney diseases as well as the socio-demographic index in Japan for the years of 1990–2016 were extracted from the Global Burden of Disease 2017 study^(13)^. Detailed information regarding the estimation of DALY and socio-demographic index have been presented elsewhere^(13,14)^. Data extraction and analysis were performed by sex (male, female and sex combined) and age group (20–49 years old, 50–69 years old, ≥70 years old and all ages). We followed the age categorisation scheme used in the Global Burden of Disease study to guarantee comparability. Our study did not use data for the 0–19-year-old age group because of the lack of risk predictor data (see below).

#### Behavioural and metabolic risk predictor, 1990–2016

We extracted the following predictors from Japan’s National Health and Nutrition Survey for 1990–2016: the proportion of those who consume <100 g (per day) of fruit and the prevalence of current smokers, current alcohol drinkers (consumption of 180·39 ml or more of alcoholic beverages for three or more days a week) and obesity for each sex- and age-group. In the current study, obesity was defined as BMI of ≥ 25 kg/m^2^ based on the Japanese Society for the Study of Obesity^(15,16)^. The National Health and Nutrition Survey is an annual and national representative household survey conducted by the Japanese Ministry of Health, Labour and Welfare to clarify dietary habits, nutrition intake and lifestyle^(17)^. The intake of nutrients, foods and alcohol was estimated based on the dietary record and the corresponding food composition list in the Standard Tables of Food Composition in Japan (sixth revised edition as at 2016)^(18)^. Since the lifestyle questionnaire, which was used to estimate the prevalence of smoking and alcohol consumption, was not conducted for the population aged less than 20 years old, only those aged ≥20 years old were considered in the current study.

### Statistical analysis and scenarios

#### Autoregressive integrated moving average model for the prediction of 2017–2040

The autoregressive integrated moving average model was used to predict the future values of the DALY rates and the risk predictors discussed above. The autoregressive integrated moving average is a statistical model that leverages time series data to forecast future trends by incorporating the past information. The past information is explicitly incorporated into the model with time lags, which allows for flexible modelling strategy to predict the future values. The autoregressive integrated moving average model combines a moving average model, which controls the dependent relationship between an observation and some lagged observations, with an autoregression model, which controls the dependency between an observation and a residual error, to model the temporal dependence over the time span using the shift and lag of historical information.

The autoregressive integrated moving average model requires the specification three parameters *p*, *d* and *q*, which is denoted as ARIMA (*p, d, q*) and generally given by(1)
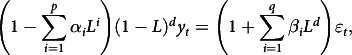

where 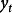 is the outcome of interest (i.e. each risk predictor), 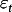 is an (white noise) error term with constant intensity of 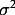 at time 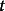 , 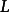 is a time lag operator defined as 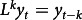 and 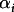 s and 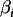 s are the coefficient parameters^(19)^. Before fitting each model, the stationarity of the time series was tested by Dickey–Fuller test^(19)^. If non-stationary was plausible, we transformed the data to satisfy the stationarity by taking a suitable difference between time points with order *d*. The autocorrelation function and partial autocorrelation function were used to estimate the stationary status and to decide the grid search range for the parameters in the models. The model parameters were estimated by maximum likelihood method. Akaike’s Information Criterion was calculated to select the best model with the parameters *p, d* and *q*.

We applied a two-step approach to predict the future DALY rates. The first step was to independently predict the future values of each predictor from 2017 to 2040 using equation (1), and then the second step was to predict the log-scaled DALY rate by using the following equation (2) after plugging the predicted values of the above predictors into 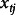 :(2)
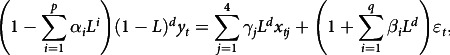

where 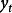 is the DALY rate at time 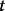 , 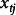 is the value of 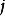 th risk predictor at time 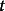 (i.e. they are predicted in the first step) and 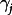 is a coefficient for the 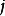 th risk predictor. All analyses were conducted by R (version 3.6.1). Using the maximum likelihood method, the parameters in equations (1) and (2) were separately estimated by disease, age and sex categories.

#### Future scenarios for the proportion of those who consume <100 g of fruit/d

We assumed four future scenarios to evaluate the impact of change in the proportion of those who consume <100 g of fruit/d on the DALY rates for the three diseases (neoplasms, cardiovascular diseases and diabetes and kidney diseases) from 2017 to 2040 in Japan: one reference and three alternative scenarios (best, moderate and constant scenarios). The reference prediction assumed that the current trend is maintained. The future proportion was predicted by the autoregressive integrated moving average model defined in equation (1). The best scenario assumed that the goal defined in Health Japan 21 (i.e. the proportion of those who consume <100 g of fruit/d is <30 %) is achieved in 2023 and the proportion is kept constant afterwards^(7)^. The moderate scenario assumed the goal defined in Health Japan 21 is achieved in 2040 instead of 2023, assuming a monotonic decrease function from 2017 to 2040. The constant scenario is a scenario in which the latest proportion (i.e. the value at 2016) is applied constantly from 2017 to 2040.

By plugging these assumed scenario values into equation (2) as predictors with other risk predictors, the final prediction values of DALY rate for these scenarios through 2040 can be obtained. It should be noted that the proportion consuming <100 g of fruit/d in 2040 is the same for the best and the moderate scenarios (i.e. both scenarios assumed that the proportions are <30 % at 2040), and the projected DALY rates converge mathematically to the same values in 2040 while the prediction trajectories until 2039 are different.

## Results

Table 1 shows the socio-demographic index and behavioural and metabolic risk predictors by sex- and age-groups from 1990 to 2016. While the prevalence of current smokers and alcohol drinkers has declined since 1990, the socio-demographic index and the prevalence of obesity have increased.

**Table 1:** Sex- and age group-specific disability-adjusted life years (DALY) rate, socio-demographic index, behavioural and metabolic risk predictor data*

Year	Sex	GBD 2017 data			SDI (%)						
		All ages DALY rate per 100,000 population^(4)^				National Health and Nutrition Survey (NHNS) data					
		Neoplasms	Cardiovascular diseases	Diabetes and kidney diseases		Number of NHNS participant	Mean age	sd	Obesity (%)	Current smoker (%)	Current alcohol drinker (%)
1990	Male	5130·206	4649·616	950·842	80·3	6182	47·4	16·1	22·3	53·1	52·1
	Female	3280·867	3674·784	828·055	80·3	7025	48·7	16·8	21·8	9·7	6·1
	Both sexes combined	4190·121	4154·074	888·425	80·3	13 207	48·1	16·5	22·0	28·5	26·0
1995	Male	5831·968	4625·992	1014·659	82·3	4976	47·8	16·5	23·9	52·7	54·4
	Female	3578·775	3573·717	839·433	82·3	5766	49·0	17·3	20·9	10·7	7·4
	Both sexes combined	4685·216	4090·442	925·478	82·3	10 742	48·4	17·0	22·2	28·2	27·0
2000	Male	6185·241	4523·508	1058·045	83·4	4513	50·1	17·0	26·8	47·4	50·8
	Female	3745·152	3344·292	844·288	83·4	5149	51·7	17·5	21·3	11·5	9·0
	Both sexes combined	4940·908	3922·162	949·039	83·4	9662	50·8	17·2	23·8	27·0	27·0
2005	Male	6454·112	4646·185	1097·638	84·4	3591	52·5	17·5	28·6	39·3	36·7
	Female	3866·874	3313·558	841·733	84·4	4155	54·5	17·8	22·0	11·4	7·4
	Both sexes combined	5131·424	3964·899	966·811	84·4	7746	53·6	17·7	24·9	24·3	20·9
2010	Male	6549·874	4664·061	1228·505	85·3	3740	53·9	17·5	30·4	32·2	35·4
	Female	3954·495	3300·696	934·343	85·3	4239	55·6	17·8	21·1	8·4	7·0
	Both sexes combined	5220·232	3965·593	1077·802	85·3	7746	54·8	17·7	25·3	19·5	20·3
2016	Male	6449·688	4590·286	1235·046	86·3	12 132	56·6	17·6	31·7	30·5	34·0
	Female	3940·317	3400·755	1003·632	86·3	14 010	58·1	18·0	21·3	7·6	7·5
	Both sexes combined	5163·140	3980·416	1116·401	86·3	26 142	57·4	17·8	25·9	18·2	19·8

GBD, Global Burden of Disease study; NHNS, National Health and Nutrition Survey of Japan; SDI, socio-demographic index.

* Note that we used data for each year, but the table lists only selected years.

Table 2 shows both the *observed* (1990–2016) and *predicted* (2017–2014) fruit intake for the four scenarios. Overall, women and older age groups consumed more fruits than men and younger age groups. Note that although the proportions of those consuming <100 g of fruits/d were at similar levels for all-ages groups in 1990, the subsequent trajectories differed by age groups from 1990 to 2016. In particular, the growth rates in younger age groups were higher (47·1 % in 1990 and 81·8 % in 2016) compared with those in the rest of the population (40·9 % (50–69 years old) and 47·3 % (≥ 70 years old) in 1990 and 58·0 % (50–69 years old) and 44·9 % (≥ 70 years old) in 2016). The Pearson’s correlations between the DALY rates and fruit intake from 1990 to 2016 were statistically significant (*P* < 0·05 for all combination of the diseases and sex-specific categorisation), except for the correlation among males who had neoplasms (*P* = 0·216).

**Table 2:** Observed (1990–2016) and predicted (2017–2014) proportion (%) of those who consume <100 g of fruit/d

Age category		20–49 years												50–69 years											
Sex		Male				Female				Both sexes combined				Male				Female				Both sexes combined			
Scenario		R	1	2	3	R	1	2	3	R	1	2	3	R	1	2	3	R	1	2	3	R	1	2	3
Observed period	1990	44·1	44·1	44·1	44·1	49·8	49·8	49·8	49·8	47·1	47·1	47·1	47·1	39·6	39·6	39·6	39·6	42·0	42·0	42·0	42·0	40·9	40·9	40·9	40·9
	1995	65·8	65·8	65·8	65·8	52·6	52·6	52·6	52·6	58·9	58·9	58·9	58·9	47·3	47·3	47·3	47·3	32·7	32·7	32·7	32·7	39·6	39·6	39·6	39·6
	2000	77·8	77·8	77·8	77·8	65·6	65·6	65·6	65·6	71·4	71·4	71·4	71·4	50·9	50·9	50·9	50·9	34·9	34·9	34·9	34·9	42·6	42·6	42·6	42·6
	2005	79·7	79·7	79·7	79·7	69·2	69·2	69·2	69·2	74·2	74·2	74·2	74·2	55·9	55·9	55·9	55·9	41·0	41·0	41·0	41·0	47·8	47·8	47·8	47·8
	2010	82·4	82·4	82·4	82·4	76·3	76·3	76·3	76·3	79·2	79·2	79·2	79·2	60·3	60·3	60·3	60·3	48·3	48·3	48·3	48·3	53·9	53·9	53·9	53·9
	2016	85·1	85·1	85·1	85·1	78·9	78·9	78·9	78·9	81·8	81·8	81·8	81·8	65·1	65·1	65·1	65·1	52·0	52·0	52·0	52·0	58·0	58·0	58·0	58·0
Prediction period	2023	97·7	30·0	70·7	85·1	85·3	30·0	66·1	78·9	91·5	30·0	68·3	81·8	71·7	30·0	55·9	65·1	50·3	30·0	46·2	52·0	60·4	30·0	50·7	58·0
	2030	100·0	30·0	53·9	85·1	92·1	30·0	51·2	78·9	100·0	30·0	52·5	81·8	77·6	30·0	45·2	65·1	50·3	30·0	39·6	52·0	64·4	30·0	42·2	58·0
	2035	100·0	30·0	42·0	85·1	96·9	30·0	40·6	78·9	100·0	30·0	41·3	81·8	82·5	30·0	37·6	65·1	50·3	30·0	34·8	52·0	67·2	30·0	36·1	58·0
	2040	100·0	30·0	30·0	85·1	100·0	30·0	30·0	78·9	100·0	30·0	30·0	81·8	86·5	30·0	30·0	65·1	50·3	30·0	30·0	52·0	70·0	30·0	30·0	58·0

Age category		≥ 70 years												All ages											
Sex		Male				Female				Both sexes combined				Male				Female				Both sexes combined			
Scenario		R	1	2	3	R	1	2	3	R	1	2	3	R	1	2	3	R	1	2	3	R	1	2	3
Observed period	1990	41·7	41·7	41·7	41·7	51·1	51·1	51·1	51·1	47·3	47·3	47·3	47·3	42·4	42·4	42·4	42·4	47·4	47·4	47·4	47·4	45·0	45·0	45·0	45·0
	1995	43·8	43·8	43·8	43·8	42·4	42·4	42·4	42·4	43·0	43·0	43·0	43·0	57·3	57·3	57·3	57·3	44·7	44·7	44·7	44·7	50·5	50·5	50·5	50·5
	2000	45·6	45·6	45·6	45·6	41·4	41·4	41·4	41·4	43·1	43·1	43·1	43·1	62·9	62·9	62·9	62·9	50·0	50·0	50·0	50·0	56·0	56·0	56·0	56·0
	2005	41·5	41·5	41·5	41·5	38·2	38·2	38·2	38·2	39·6	39·6	39·6	39·6	63·0	63·0	63·0	63·0	51·3	51·3	51·3	51·3	56·7	56·7	56·7	56·7
	2010	46·2	46·2	46·2	46·2	42·6	42·6	42·6	42·6	44·2	44·2	44·2	44·2	66·0	66·0	66·0	66·0	57·3	57·3	57·3	57·3	61·3	61·3	61·3	61·3
	2016	46·3	46·3	46·3	46·3	43·8	43·8	43·8	43·8	44·9	44·9	44·9	44·9	67·0	67·0	67·0	67·0	58·4	58·4	58·4	58·4	62·3	62·3	62·3	62·3
Prediction period	2020	45·3	30·0	42·1	46·3	42·4	30·0	40·2	43·8	42·8	30·0	41·0	44·9	67·0	30·0	57·4	67·0	56·6	30·0	51·0	58·4	66·3	30·0	53·9	62·3
	2023	45·3	30·0	37·1	46·3	42·4	30·0	36·0	43·8	42·8	30·0	36·5	44·9	67·0	30·0	46·1	67·0	56·6	30·0	42·3	58·4	70·2	30·0	44·1	62·3
	2035	45·3	30·0	33·5	46·3	42·4	30·0	33·0	43·8	42·8	30·0	33·2	44·9	67·0	30·0	38·0	67·0	56·6	30·0	36·2	58·4	73·0	30·0	37·0	62·3
	2040	45·3	30·0	30·0	46·3	42·4	30·0	30·0	43·8	42·8	30·0	30·0	44·9	67·0	30·0	30·0	67·0	56·6	30·0	30·0	58·4	75·8	30·0	30·0	62·3

R, reference scenario; 1, best scenario; 2, moderate scenario; 3, constant scenario.

### Future trends of disability-adjusted life years rate for neoplasms, cardiovascular diseases and diabetes and kidney diseases

#### Neoplasms

**Fig. 1:**
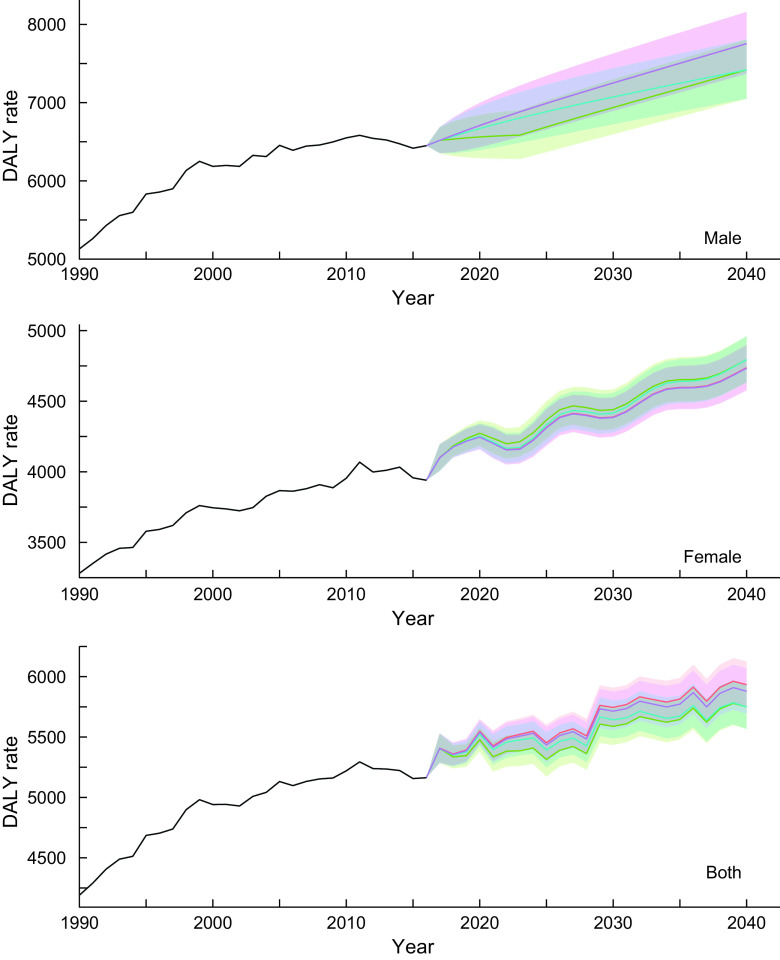
Observed and projected all ages disability-adjusted life years (DALY) rate (per 100 000) for neoplasms for reference forecast and three alternative scenarios, 1990–2040: male, female and both sexes combined. 1: best scenario, 2: moderate scenario, 3: constant scenario. It is important to note that the y-axis scales are different for each panel in order to make the differences between scenarios easier to understand. Detailed values are shown in online Supplemental Table 3. 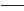, Observed; 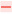, Reference; 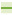, Scenario 1; 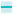, Scenario 2; 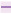, Scenario 3

The estimated parameters (*p,d, q*) in each autoregressive integrated moving average model defined by equation (2) and their Akaike Information Criteria are provided in online Supplemental Table 1. Figure 1 shows the trend of DALY rates for neoplasms for all-ages group by sex and scenario. Detailed and age group-specific DALY rates are provided in online Supplemental Figures 1–3 (1: 20–49 years old, 2: 50–69 years old and 3: ≥ 70 years old, respectively). Online Supplemental Figures 1–3 show that the DALY rates have decreasing trends through 2040 in every age- and sex-group (except for men aged ≥70 years old) while the total population estimate shows an increasing trend. This discrepancy between the age group specific and total population estimates suggests that the latter was greatly affected by population ageing. In the reference scenario, the greatest decline in DALY rates was expected in the 50–69-year-old age group, with declines of 14·9, 8·9 and 13·6 % for men, women and sex-combined groups, respectively, from 2016 to 2040. Exact predicted values of DALY rates in 2040 for 20–49 years old, 50–69 years old, ≥ 70 years old and all-ages group and for men, women and both sex-combined groups and for all scenarios are presented in online Supplemental Table 2.

#### Cardiovascular diseases

**Fig. 2:**
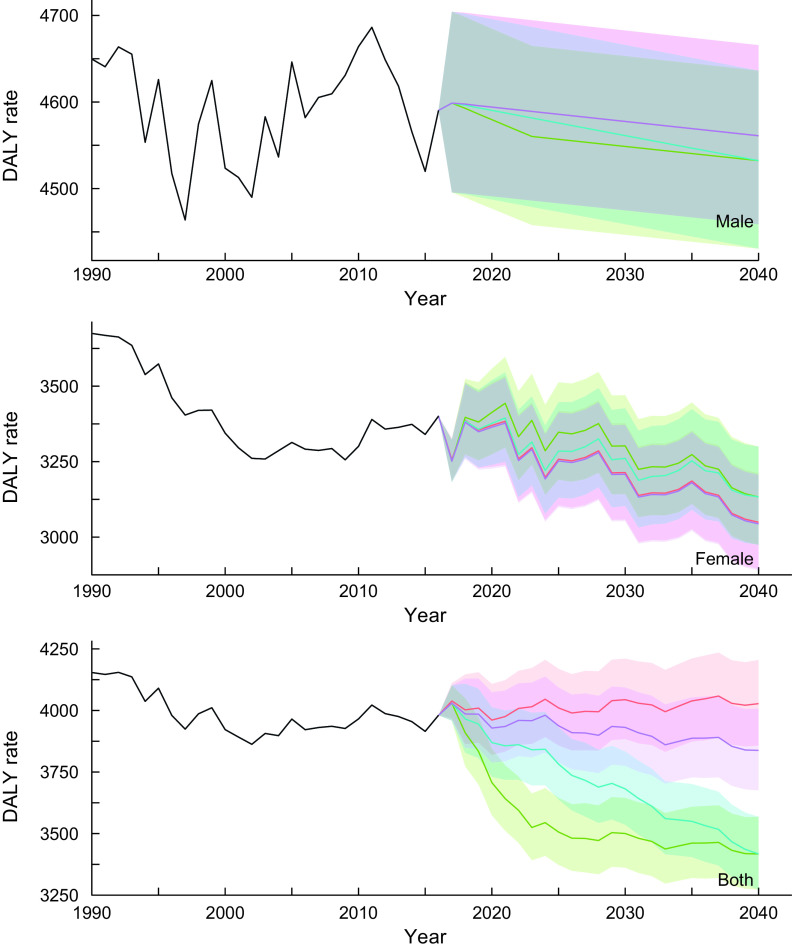
Observed and projected all ages disability-adjusted life years (DALY) rate (per 100 000) for cardiovascular diseases for reference forecast and three alternative scenarios, 1990–2040: male, female and both sexes combined. 1: best scenario, 2: moderate scenario, 3: constant scenario. It is important to note that the y-axis scales are different for each panel in order to make the differences between scenarios easier to understand. Detailed values are shown in Supplemental Table 4. 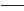, Observed; 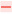, Reference; 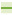, Scenario 1; 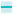, Scenario 2; 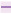, Scenario 3

Figure 2 shows the trends of DALY rates for cardiovascular diseases for all-ages group by sex and scenarios. More detailed and age group-specific DALY rates are provided in online Supplemental Figures 4–6 (4: 20–49 years old, 5: 50–69 years old and 6: ≥70 years old, respectively). The DALY rates continued to decline through 2040 regardless of the scenarios, sex- and age-groups. In the reference scenario, the greatest decline in DALY rates was expected in the ≥70-year-old age group, with average declines of 41·7, 42·3 and 39·3 % for male, female and sex-combined groups from 2016 to 2040, respectively. Additionally, the DALY rates in sex- and all-ages groups show remarkable differences between the scenarios with nonoverlapping 95 % prediction intervals (PI): DALY rates (95 % PIs) in 2040 were 4027·7 (3857·5, 4205·3) and 3837·9 (3675·7, 4007·1) for reference and constant scenarios, respectively, while DALY rates (95 % PIs) in 2040 were 3417·3 (3272·9, 3568·0) for better and moderate scenarios. Similar results were observed by age groups, particularly in men and sex-combined groups aged 20–49 years.

#### Diabetes and kidney diseases

**Fig. 3:**
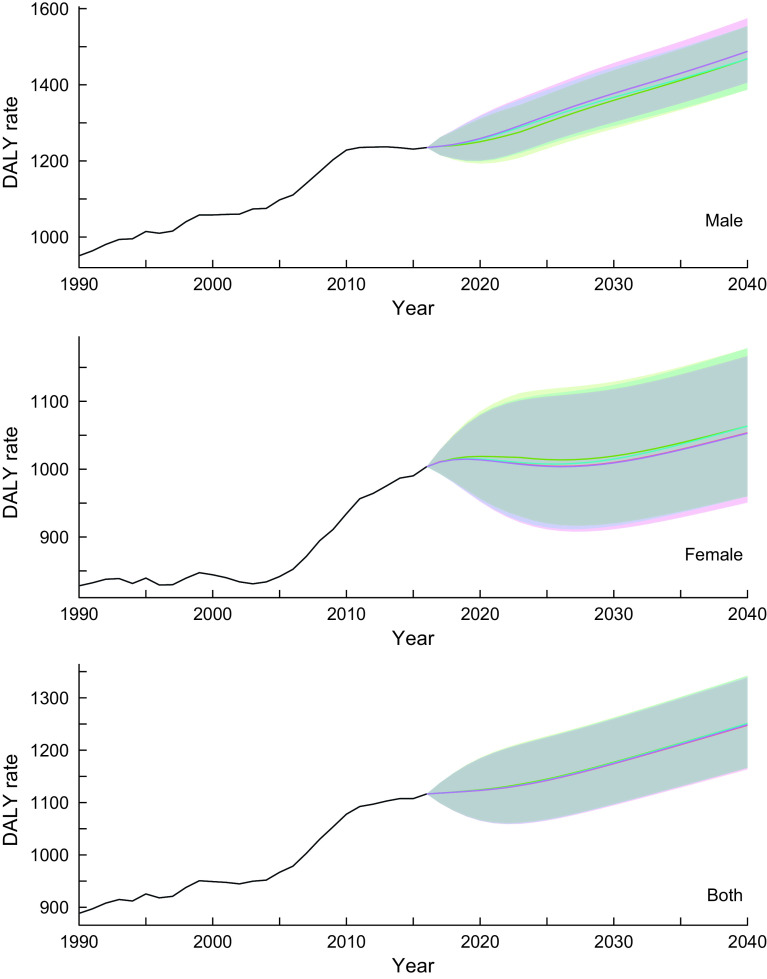
Observed and projected all ages disability-adjusted life years (DALY) rate (per 100 000) for diabetes and kidney disease for reference forecast and three alternative scenarios, 1990–2040: male, female and both sexes combined. 1: best scenario, 2: moderate scenario, 3: constant scenario. It is important to note that the y-axis scales are different for each panel in order to make the differences between scenarios easier to understand. Detailed values are shown in online Supplemental Table 5. 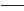, Observed; 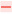, Reference; 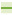, Scenario 1; 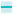, Scenario 2; 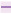, Scenario 3

Figure 3 shows the trends of DALY rates for diabetes and kidney diseases for all-ages group by sex and scenarios. More detailed and age group-specific DALY rates are provided in online Supplemental Figures 7–9 (7: 20–49 years old, 8: 50–69 years old and 9: ≥70 years old, respectively). As with neoplasms’ trend, the age group-specific DALY rates show a decreasing trend, while the DALY rates in all-ages group show an increasing trend, also suggesting that the effects of the population ageing on future DALY rate of diabetes and kidney diseases might be significant. Except for males aged 50–69 years, there was no remarkable difference in DALY rates between the scenarios with overlapping PI. In the reference scenario, the greatest decline in DALY rate was expected in the ≥70-year-old age group, with average declines of 13·4, 22·1 and 20·5 % for male, female and sex-combined groups from 2016 to 2040.

## Discussion

To optimize long-term medical investment and policy implementation, it is important to understand the future trajectories of health and drivers of health at the population level. In the current study, we predicted a set of cause-, sex- and age group-specific DALY rates for chronic diseases that have previously been associated with low fruit intake by assessing four future scenarios of the proportion of the Japanese population that consume <100 g of fruit/d. Our analyses indicate that increasing fruit intake may have a potential to reduce disease burden and thereby contribute to addressing the major policy issue of increasing healthcare costs due to the ageing society and the extension of life expectancy^(11)^.

Overall, the DALY rates in the reference scenario were predicted to increase for neoplasms and decrease for cardiovascular diseases and diabetes and kidney diseases through 2040 (except for the sex-combined group for cardiovascular diseases, which showed the stable trend). Similarly, the age group-specific DALY rates for these three causes were predicted to decrease (except for male aged ≥70-year-old group, which shows a stable trend). Importantly, in all sex- and age-groups and among men aged 20–49 years, the predicted DALY rates attributable to cardiovascular diseases between four assumed scenarios did not overlap with one another greatly, suggesting that there is a significant potential for reducing the burden of cardiovascular diseases by increasing fruit intake at the population level. Our findings are in accordance with previous studies in Japan providing a clear inverse association of fruit intake and risk of cardiovascular diseases and diabetes and kidney diseases. For example, Takachi *et al*.^(20)^, who conducted a survival analysis of participants aged 45–75 years old in several cohorts in Japan from 1990 to 2003, showed an inverse relationship between fruit intake and risk of cancer and cardiovascular diseases. Kurotani *et al*.^(21)^ used the same data set and conducted a logistic regression analysis to indicate the association between high fruit intake and low risk of diabetes. Nagura *et al.* (2009) conducted a survival analysis with a different multi-cohort data set of participants aged 45–79 years from 1988 to 2003^(22)^ to reach the same conclusion for mortality risk on cardiovascular diseases.

Fruits are rich sources of potassium, folate, fibre and antioxidants, including vitamin C, *β*-carotene and flavonoids^(23)^. Previous randomised controlled trials showed that increasing fruit (and vegetable) intake can reduce blood pressure as well as increase urinary K excretion^(24)^. Since raised blood pressure is a major risk factor for cardiovascular diseases, the effect of fruit to lower blood pressure could be one of the major biological mechanisms that leads to a reduced risk of cardiovascular diseases^(25)^. Limited evidence provides that as part of a healthy diet low in fat, sugars and salt/Na; fruits (and vegetables) may prevent weight gain and reduce the risk of obesity, a risk factor for the three diseases of our interest^(21,26)^. Additionally, the previous studies suggest the potential impact of fruit intake on shifting towards healthier and more sustainable diets globally^(27,28)^ as well as the effect for reducing the risk of cardiovascular diseases^(20,22,27,28)^. Our models do not support the notion that a substantial benefit from increased fruit intake can be obtained with respect to neoplasms and diabetes and kidney diseases (i.e. prediction trajectories among most scenarios have overlapping prediction intervals). It suggests that there may be other risk factors at play, including physical activity and social determinants of health such as physical environment and social relationships with other persons, all of which have more significant impact on the three disease groups of interest^(2,29–31)^. It is our ongoing study to merge our data set with other data sources that include possible confounders to adjust for the statistical association between the DALY rates and fruit intake.

The ten-year health promotion guideline ‘Health Japan 21 (second phase)’ has focused on nation-wide campaigns to raise awareness and to induce behavioural change for increasing fruit intake. At the same time it has also tried to encourage the food industry and the Japanese society at large to promote the consumption of a balanced diet, including the adequate amount of fruit intake. As we have shown, the proportion of those who consume <100 g of fruit/d in Japan has been increasing since 1990 and reached 62·3 % in 2016^(4,5,10)^. This is far from the target of Health Japan 21. WHO recommends several effective actions for policy-makers to create a healthy food environment; for example, by applying school interventions that encourage children to adopt and maintain a healthy diet and exploring economic incentives or disincentives, including taxation and subsidies, to promote a healthy diet^(8,32,33)^. Since low fruit intake is a modifiable factor application of the interventions presented in WHO’s guidelines, clinical and political actions are urgently demanded to prevent disease, prolong healthy life and efficiently maintain and improve population health^(11)^.

The strengths of our study lie in the fact that we use the best available data that represent the Japanese population’s dietary pattern over time with a flexible time series modelling approach. Simple models as ours have advantages in allowing for a prompt exploration of dietary risk factors and relevant disease burden forecasts. Our study, however, has limitations. First, several risk predictors, which were not included in our models, could change the prediction results. For example, blood pressure, which is known to be associated with cardiovascular diseases and diabetes and kidney diseases^(34–36)^, was not included in our models because of data availability. We welcome re-evaluation of our results by using more detailed data of risk predictors. Second, in Japan, health outcomes are largely explained by health system performance^(11)^. The prediction may be influenced by these health drivers more than by individual risk factors. Third, while DALY is a well-established population health measure that is widely used as an important benchmark for making health policy decision, it has many limitations as discussed elsewhere^(37)^. In particular, DALY cannot clearly answer the question about how much investment is required to reduce the burden of disease. Finally, our study is also subject to similar limitations as other studies concerning dietary patterns^(38,39)^. In the National Health and Nutrition Survey, dietary intake was estimated based on self-report and recorded in a single day, which might not represent a long-term and/or seasonal dietary pattern. Additionally, data based on self-reporting may be subject to bias, such as the social-desirability-response bias^(17,40)^ and measurement error in comparison to medical records or laboratory-confirmed data.

In conclusion, our prediction results show that higher fruit intake is associated with lower disease burden in Japan. Further research is required to assess which policies and interventions can be used to achieve an increase in fruit intake as modelled in the scenarios of the current study. In addition, our analytical framework using autoregressive integrated moving average model might be useful for other countries by providing information for assessing how the control of fruit intake can impact the population health.

## Supplementary material

For supplementary material accompanying this paper visit https://doi.org/10.1017/S1368980020004541. click here to view supplementary material
