# Supplementary material for: Prediction of disability-adjusted life years for diseases due to low fruit intake in 2017–2040 in Japan
Source: Public Health Nutr. 2020 Nov 13;24(10):3156–66. doi: 10.1017/S1368980020004541 (PMC9884779; doi:10.1017/S1368980020004541)
Supplement: Supplementary file 1 [file S1368980020004541sup.zip › urn_cambridge.org_id_binary_20210107050814643-0358_S1368980020004541sup001.docx]

**Supplemental Table 1: Estimated sets of parameters in autoregressive integrated moving average model (ARIMA(*p,d,q*)) and Akaike Information Criteria (AIC)**

| Disease | Age group | Sex | *p* | *d* | *q* | AIC |
| --- | --- | --- | --- | --- | --- | --- |
| Neoplasms | 20–49 | Male | 6 | 0 | 0 | -120.058 |
|  |  | Female | 6 | 0 | 0 | -128.3929 |
|  |  | Both sexes combined | 7 | 0 | 0 | -132.223 |
|  | 50–69 | Male | 6 | 0 | 0 | -135.0067 |
|  |  | Female | 4 | 0 | 0 | -144.1677 |
|  |  | Both sexes combined | 8 | 0 | 3 | -150.1343 |
|  | ≥70 | Male | 5 | 0 | 1 | -139.0884 |
|  |  | Female | 5 | 0 | 0 | -156.7754 |
|  |  | Both sexes combined | 4 | 0 | 0 | -147.931 |
|  | All ages | Male | 1 | 0 | 0 | -140.9591 |
|  |  | Female | 2 | 0 | 6 | -154.2407 |
|  |  | Both sexes combined | 8 | 0 | 0 | -148.1379 |
| Cardiovascular diseases | 20–49 | Male | 7 | 0 | 0 | -133.9074 |
|  |  | Female | 9 | 0 | 0 | -139.9755 |
|  |  | Both sexes combined | 7 | 0 | 0 | -150.0904 |
|  | 50–69 | Male | 7 | 0 | 0 | -144.3471 |
|  |  | Female | 8 | 0 | 3 | -155.3689 |
|  |  | Both sexes combined | 6 | 0 | 0 | -149.6124 |
|  | ≥70 | Male | 7 | 0 | 0 | -127.6131 |
|  |  | Female | 5 | 0 | 6 | -141.3358 |
|  |  | Both sexes combined | 4 | 0 | 0 | -137.183 |
|  | All ages | Male | 0 | 0 | 0 | -149.615 |
|  |  | Female | 6 | 0 | 3 | -147.5468 |
|  |  | Both sexes combined | 4 | 0 | 6 | -156.9722 |
| Diabetes and kidney diseases | 20–49 | Male | 2 | 0 | 0 | -158.19 |
|  |  | Female | 4 | 0 | 2 | -184.5957 |
|  |  | Both sexes combined | 2 | 0 | 0 | -167.5673 |
|  | 50–69 | Male | 6 | 0 | 0 | -165.9671 |
|  |  | Female | 4 | 0 | 5 | -176.7471 |
|  |  | Both sexes combined | 6 | 0 | 0 | -167.4724 |
|  | ≥70 | Male | 4 | 0 | 0 | -149.5586 |
|  |  | Female | 4 | 0 | 8 | -163.7399 |
|  |  | Both sexes combined | 4 | 0 | 0 | -162.1955 |
|  | All ages | Male | 3 | 0 | 0 | -153.3324 |
|  |  | Female | 2 | 0 | 0 | -155.7727 |
|  |  | Both sexes combined | 2 | 0 | 0 | -157.5894 |

**Supplementary table 2: Observed and projected DALYs rate (per 100,000) in GBD 2016, and in the 2040 reference forecast and three alternative scenarios**

| Disease | Age group | Sex | 2016 [5] | 2040 forecast | 2040 better and moderate | 2040 constant |
| --- | --- | --- | --- | --- | --- | --- |
|  |  |  |  | (95% PIs) | scenarios (95% PIs)* | scenario (95% PIs) |
| Neoplasms | 15–49 | Male | 929.4 | 593.7 (535.3, 658.5) | 496.2 (447.3, 550.3) | 571.4 (515.2, 633.8) |
|  |  | Female | 1,137.0 | 922.6 (854.2, 996.5) | 891.3 (825.2, 962.6) | 913.0 (845.4, 986.1) |
|  |  | Both sexes combined | 1,031.4 | 738.2 (681.7, 799.3) | 698.2 (644.8, 756.0) | 727.5 (671.9, 787.8) |
|  | 50–69 | Male | 9,860.2 | 8388.9 (7738.8, 9093.5) | 7244.6 (6683.2, 7853.2) | 7935.3 (7320.4, 8601.8) |
|  |  | Female | 5,469.0 | 4981.6 (4751.4, 5223.0) | 4962.7 (4733.4, 5203.2) | 4983.2 (4753.0, 5224.7) |
|  |  | Both sexes combined | 7,636.3 | 6599.3 (5825.1, 7476.5) | 6599.5 (5825.3, 7476.7) | 6599.4 (5825.2, 7476.5) |
|  | ≥70 | Male | 20,818.2 | 21093.9 (18681.5, 23817.7) | 21270.3 (18837.8, 24016.9) | 21082.2 (18671.2, 23804.5) |
|  |  | Female | 9,342.7 | 8407.1 (8169.0, 8652.1) | 8431.5 (8192.7, 8677.2) | 8404.3 (8166.3, 8649.2) |
|  |  | Both sexes combined | 14,102.1 | 13459.9 (12389, 14623.4) | 13457.0 (12386.3, 14620.2) | 13460.4 (12389.4, 14623.9) |
|  | All | Male | 6,449.7 | 7754.7 (7368.7, 8161.0) | 7419.5 (7050.1, 7808.2) | 7754.7 (7368.7, 8161.0) |
|  |  | Female | 3,940.3 | 4737.5 (4579.0, 4901.5) | 4794.4 (4634.0, 4960.4) | 4733.8 (4575.4, 4897.7) |
|  |  | Both sexes combined | 5,163.1 | 5933.4 (5746.4, 6126.5) | 5749.0 (5567.8, 5936.1) | 5878.6 (5693.3, 6069.9) |
| Cardiovascular diseases | 15–49 | Male | 1,052.2 | 832.8 (795.6, 871.8) | 671.3 (641.3, 702.7) | 795.4 (759.9, 832.6) |
|  |  | Female | 501.0 | 359.5 (337.1, 383.3) | 377.8 (354.3, 402.8) | 364.9 (342.2, 389.1) |
|  |  | Both sexes combined | 781.2 | 617.3 (593.0, 642.7) | 565.3 (543.0, 588.5) | 603.4 (579.6, 628.2) |
|  | 50–69 | Male | 5,790.2 | 4265.2 (4086.7, 4451.4) | 4039.3 (3870.3, 4215.7) | 4178.1 (4003.3, 4360.5) |
|  |  | Female | 2,600.1 | 1647.6 (1533.9, 1769.6) | 1669.8 (1554.6, 1793.4) | 1645.7 (1532.2, 1767.6) |
|  |  | Both sexes combined | 4,174.7 | 2953.3 (2809.7, 3104.2) | 3010.0 (2863.7, 3163.8) | 2970.2 (2825.8, 3122.0) |
|  | ≥70 | Male | 15,884.6 | 9265.5 (8652.7, 9921.7) | 9316.1 (8699.9, 9975.8) | 9262.2 (8649.6, 9918.1) |
|  |  | Female | 11,594.4 | 6694.2 (6089.1, 7359.4) | 6747.1 (6137.2, 7417.6) | 6688.0 (6083.5, 7352.6) |
|  |  | Both sexes combined | 13,373.8 | 8113.1 (7460.3, 8822.9) | 8088.0 (7437.3, 8795.7) | 8117.1 (7464.0, 8827.3) |
|  | All | Male | 4,590.3 | 4561.0 (4458.7, 4665.7) | 4532.3 (4430.6, 4636.3) | 4561.0 (4458.7, 4665.7) |
|  |  | Female | 3,400.8 | 3049.2 (2895.0, 3211.7) | 3133.1 (2974.6, 3300.0) | 3043.8 (2889.9, 3206.0) |
|  |  | Both sexes combined | 3,980.4 | 4027.7 (3857.5, 4205.3) | 3417.3 (3272.9, 3568.0) | 3837.9 (3675.7, 4007.1) |
| Diabetes and kidney diseases | 15–49 | Male | 406.6 | 367.7 (330.4, 409.2) | 358.0 (321.7, 398.4) | 365.6 (328.5, 406.9) |
|  |  | Female | 301.1 | 271.9 (244.1, 302.8) | 276.9 (248.6, 308.5) | 273.4 (245.4, 304.5) |
|  |  | Both sexes combined | 354.7 | 325.4 (288.6, 366.8) | 321.0 (284.7, 361.8) | 324.2 (287.6, 365.5) |
|  | 50–69 | Male | 1,769.5 | 1625.1 (1552.4, 1701.2) | 1489.9 (1423.2, 1559.7) | 1572.4 (1502.1, 1646.1) |
|  |  | Female | 1,109.4 | 898.0 (782.0, 1031.3) | 904.1 (787.3, 1038.3) | 897.5 (781.6, 1030.7) |
|  |  | Both sexes combined | 1,435.2 | 1246.4 (1155.2, 1344.7) | 1249.9 (1158.5, 1348.5) | 1247.4 (1156.2, 1345.8) |
|  | ≥70 | Male | 3,585.3 | 3103.8 (2907.7, 3313.1) | 3133.7 (2935.7, 3345.0) | 3101.8 (2905.9, 3311.0) |
|  |  | Female | 2,721.2 | 2120.6 (1972.7, 2279.6) | 2128.2 (1979.7, 2287.7) | 2119.7 (1971.9, 2278.6) |
|  |  | Both sexes combined | 3,079.6 | 2449.7 (2314.4, 2592.8) | 2448.9 (2313.7, 2592.0) | 2449.8 (2314.6, 2593.0) |
|  | All | Male | 1,235.0 | 1487.9 (1405.6, 1574.9) | 1468.4 (1387.2, 1554.3) | 1487.9 (1405.6, 1574.9) |
|  |  | Female | 1,003.6 | 1053.6 (951.0, 1167.3) | 1063.5 (959.9, 1178.3) | 1053.0 (950.4, 1166.6) |
|  |  | Both sexes combined | 1,116.4 | 1247.6 (1162.9, 1338.4) | 1251.0 (1166.1, 1342.1) | 1248.6 (1163.9, 1339.5) |

GBD: Global Burden of Disease study; PIs: prediction intervals. * Because the proportion of those who consume less than 100 g of fruit as of 2040 is the same by definition in the best and moderate scenarios, the projected DALYs rate converged mathematically to the same values.

**Supplementary table 3:** **Observed and projected all ages DALYs rate per 100,000 (95% PIs) for neoplasms for reference forecast and three alternative scenarios, 1990–2040: male, female and both sexes combined**

|  | Neoplasms | | | | | | | | | | | |
| --- | --- | --- | --- | --- | --- | --- | --- | --- | --- | --- | --- | --- |
|  | Male | | | | Female | | | | Both sexed combined | | | |
| Year | Reference | Scenario 1 | Scenario 2 | Scenario 3 | Reference | Scenario 1 | Scenario 2 | Scenario 3 | Reference | Scenario 1 | Scenario 2 | Scenario 3 |
| 1990 | 5130.21 | 5130.21 | 5130.21 | 5130.21 | 3280.87 | 3280.87 | 3280.87 | 3280.87 | 4190.12 | 4190.12 | 4190.12 | 4190.12 |
| 1991 | 5261.63 | 5261.63 | 5261.63 | 5261.63 | 3350.52 | 3350.52 | 3350.52 | 3350.52 | 4289.94 | 4289.94 | 4289.94 | 4289.94 |
| 1992 | 5429.23 | 5429.23 | 5429.23 | 5429.23 | 3416.97 | 3416.97 | 3416.97 | 3416.97 | 4405.90 | 4405.90 | 4405.90 | 4405.90 |
| 1993 | 5555.70 | 5555.70 | 5555.70 | 5555.70 | 3457.98 | 3457.98 | 3457.98 | 3457.98 | 4488.65 | 4488.65 | 4488.65 | 4488.65 |
| 1994 | 5598.21 | 5598.21 | 5598.21 | 5598.21 | 3463.89 | 3463.89 | 3463.89 | 3463.89 | 4512.26 | 4512.26 | 4512.26 | 4512.26 |
| 1995 | 5831.97 | 5831.97 | 5831.97 | 5831.97 | 3578.78 | 3578.78 | 3578.78 | 3578.78 | 4685.22 | 4685.22 | 4685.22 | 4685.22 |
| 1996 | 5857.00 | 5857.00 | 5857.00 | 5857.00 | 3591.87 | 3591.87 | 3591.87 | 3591.87 | 4703.80 | 4703.80 | 4703.80 | 4703.80 |
| 1997 | 5899.00 | 5899.00 | 5899.00 | 5899.00 | 3619.85 | 3619.85 | 3619.85 | 3619.85 | 4738.26 | 4738.26 | 4738.26 | 4738.26 |
| 1998 | 6132.94 | 6132.94 | 6132.94 | 6132.94 | 3709.72 | 3709.72 | 3709.72 | 3709.72 | 4898.33 | 4898.33 | 4898.33 | 4898.33 |
| 1999 | 6249.74 | 6249.74 | 6249.74 | 6249.74 | 3760.82 | 3760.82 | 3760.82 | 3760.82 | 4981.10 | 4981.10 | 4981.10 | 4981.10 |
| 2000 | 6185.24 | 6185.24 | 6185.24 | 6185.24 | 3745.15 | 3745.15 | 3745.15 | 3745.15 | 4940.91 | 4940.91 | 4940.91 | 4940.91 |
| 2001 | 6197.35 | 6197.35 | 6197.35 | 6197.35 | 3737.00 | 3737.00 | 3737.00 | 3737.00 | 4942.10 | 4942.10 | 4942.10 | 4942.10 |
| 2002 | 6185.42 | 6185.42 | 6185.42 | 6185.42 | 3723.78 | 3723.78 | 3723.78 | 3723.78 | 4928.91 | 4928.91 | 4928.91 | 4928.91 |
| 2003 | 6325.79 | 6325.79 | 6325.79 | 6325.79 | 3746.29 | 3746.29 | 3746.29 | 3746.29 | 5008.43 | 5008.43 | 5008.43 | 5008.43 |
| 2004 | 6310.98 | 6310.98 | 6310.98 | 6310.98 | 3826.99 | 3826.99 | 3826.99 | 3826.99 | 5041.73 | 5041.73 | 5041.73 | 5041.73 |
| 2005 | 6454.11 | 6454.11 | 6454.11 | 6454.11 | 3866.87 | 3866.87 | 3866.87 | 3866.87 | 5131.42 | 5131.42 | 5131.42 | 5131.42 |
| 2006 | 6391.04 | 6391.04 | 6391.04 | 6391.04 | 3862.41 | 3862.41 | 3862.41 | 3862.41 | 5097.65 | 5097.65 | 5097.65 | 5097.65 |
| 2007 | 6444.56 | 6444.56 | 6444.56 | 6444.56 | 3880.07 | 3880.07 | 3880.07 | 3880.07 | 5132.23 | 5132.23 | 5132.23 | 5132.23 |
| 2008 | 6458.44 | 6458.44 | 6458.44 | 6458.44 | 3908.75 | 3908.75 | 3908.75 | 3908.75 | 5153.15 | 5153.15 | 5153.15 | 5153.15 |
| 2009 | 6497.79 | 6497.79 | 6497.79 | 6497.79 | 3886.83 | 3886.83 | 3886.83 | 3886.83 | 5160.63 | 5160.63 | 5160.63 | 5160.63 |
| 2010 | 6549.87 | 6549.87 | 6549.87 | 6549.87 | 3954.50 | 3954.50 | 3954.50 | 3954.50 | 5220.23 | 5220.23 | 5220.23 | 5220.23 |
| 2011 | 6582.33 | 6582.33 | 6582.33 | 6582.33 | 4068.39 | 4068.39 | 4068.39 | 4068.39 | 5294.11 | 5294.11 | 5294.11 | 5294.11 |
| 2012 | 6543.22 | 6543.22 | 6543.22 | 6543.22 | 3998.53 | 3998.53 | 3998.53 | 3998.53 | 5239.03 | 5239.03 | 5239.03 | 5239.03 |
| 2013 | 6522.70 | 6522.70 | 6522.70 | 6522.70 | 4010.68 | 4010.68 | 4010.68 | 4010.68 | 5235.09 | 5235.09 | 5235.09 | 5235.09 |
| 2014 | 6473.90 | 6473.90 | 6473.90 | 6473.90 | 4032.92 | 4032.92 | 4032.92 | 4032.92 | 5222.61 | 5222.61 | 5222.61 | 5222.61 |
| 2015 | 6417.40 | 6417.40 | 6417.40 | 6417.40 | 3957.22 | 3957.22 | 3957.22 | 3957.22 | 5156.19 | 5156.19 | 5156.19 | 5156.19 |
| 2016 | 6449.69 | 6449.69 | 6449.69 | 6449.69 | 3940.32 | 3940.32 | 3940.32 | 3940.32 | 5163.14 | 5163.14 | 5163.14 | 5163.14 |
| 2017 | 6517.87 (6354.05, 6685.91) | 6517.87 (6354.05, 6685.91) | 6517.87 (6354.05, 6685.91) | 6517.87 (6354.05, 6685.91) | 4101.65 (4008.28, 4197.19) | 4098.88 (4005.57, 4194.36) | 4098.88 (4005.57, 4194.36) | 4098.88 (4005.57, 4194.36) | 5407.93 (5287.11, 5531.52) | 5405.68 (5284.9, 5529.22) | 5405.68 (5284.9, 5529.22) | 5405.68 (5284.9, 5529.22) |
| 2018 | 6582.03 (6363.97, 6807.56) | 6533.73 (6317.27, 6757.6) | 6569.39 (6351.76, 6794.49) | 6582.03 (6363.97, 6807.56) | 4178.34 (4102.59, 4255.48) | 4183.79 (4107.94, 4261.03) | 4177.23 (4101.51, 4254.36) | 4174.92 (4099.24, 4252.01) | 5359.33 (5264.12, 5456.26) | 5335.17 (5240.39, 5431.66) | 5349.84 (5254.8, 5446.6) | 5355.03 (5259.9, 5451.88) |
| 2019 | 6646.76 (6394.15, 6909.35) | 6549.56 (6300.65, 6808.31) | 6621.27 (6369.63, 6882.85) | 6646.76 (6394.15, 6909.35) | 4220.09 (4134.64, 4307.29) | 4234.78 (4149.04, 4322.29) | 4221.52 (4136.05, 4308.76) | 4216.86 (4131.48, 4304) | 5392.57 (5298.65, 5488.15) | 5346.28 (5253.17, 5441.04) | 5375.73 (5282.11, 5471.02) | 5386.17 (5292.36, 5481.64) |
| 2020 | 6708.02 (6431.16, 6996.8) | 6561.41 (6290.61, 6843.88) | 6669.46 (6394.19, 6956.58) | 6708.02 (6431.16, 6996.8) | 4249.24 (4160.98, 4339.37) | 4273 (4184.25, 4363.63) | 4252.96 (4164.63, 4343.17) | 4245.91 (4157.72, 4335.96) | 5548.31 (5448.44, 5650.01) | 5478.17 (5379.56, 5578.58) | 5523.5 (5424.08, 5624.74) | 5539.59 (5439.88, 5641.13) |
| 2021 | 6767.71 (6472.91, 7075.93) | 6571.22 (6284.98, 6870.48) | 6715.89 (6423.35, 7021.75) | 6767.71 (6472.91, 7075.93) | 4204.43 (4097.38, 4314.29) | 4236.95 (4129.06, 4347.65) | 4210.47 (4103.26, 4320.48) | 4201.17 (4094.19, 4310.94) | 5426.83 (5302.27, 5554.32) | 5336.3 (5213.82, 5461.66) | 5395.26 (5271.42, 5522) | 5416.22 (5291.9, 5543.46) |
| 2022 | 6825.53 (6517.04, 7148.62) | 6578.73 (6281.39, 6890.13) | 6760.27 (6454.73, 7080.27) | 6825.53 (6517.04, 7148.62) | 4157.91 (4052.89, 4265.65) | 4198.95 (4092.89, 4307.75) | 4166.17 (4060.94, 4274.13) | 4154.67 (4049.73, 4262.33) | 5496.44 (5365.59, 5630.49) | 5382.62 (5254.47, 5513.89) | 5457.06 (5327.14, 5590.14) | 5483.58 (5353.03, 5617.31) |
| 2023 | 6881.95 (6562.72, 7216.71) | 6584.42 (6279, 6904.71) | 6803.06 (6487.49, 7133.99) | 6881.95 (6562.72, 7216.71) | 4162.41 (4060.55, 4266.82) | 4212.42 (4109.34, 4318.09) | 4172.99 (4070.88, 4277.67) | 4159.17 (4057.39, 4263.5) | 5522.28 (5393.98, 5653.63) | 5385.79 (5260.66, 5513.89) | 5475.29 (5348.08, 5605.52) | 5507.23 (5379.28, 5638.22) |
| 2024 | 6937.11 (6609.26, 7281.22) | 6637.19 (6323.52, 6966.43) | 6844.42 (6520.95, 7183.94) | 6937.11 (6609.26, 7281.22) | 4225.1 (4107.69, 4345.88) | 4275.87 (4157.04, 4398.09) | 4238.19 (4120.41, 4359.34) | 4221.81 (4104.49, 4342.49) | 5547.95 (5414.06, 5685.14) | 5408.74 (5278.21, 5542.49) | 5493.29 (5360.72, 5629.14) | 5530.7 (5397.23, 5667.47) |
| 2025 | 6991.21 (6656.29, 7342.99) | 6688.96 (6368.52, 7025.53) | 6884.56 (6554.75, 7230.97) | 6991.21 (6656.29, 7342.99) | 4314.93 (4186.46, 4447.34) | 4366.77 (4236.76, 4500.77) | 4330.69 (4201.75, 4463.58) | 4311.57 (4183.2, 4443.87) | 5453.12 (5307.4, 5602.84) | 5314.25 (5172.24, 5460.15) | 5392.09 (5248.01, 5540.14) | 5434.08 (5288.87, 5583.27) |
| 2026 | 7044.44 (6703.59, 7402.61) | 6739.88 (6413.77, 7082.58) | 6923.66 (6588.66, 7275.69) | 7044.44 (6703.59, 7402.61) | 4387.19 (4259.8, 4518.39) | 4439.9 (4310.98, 4572.67) | 4405.65 (4277.72, 4537.4) | 4383.77 (4256.48, 4514.87) | 5533.64 (5397.78, 5672.92) | 5390.64 (5258.29, 5526.32) | 5464.31 (5330.15, 5601.84) | 5512.19 (5376.86, 5650.93) |
| 2027 | 7096.92 (6751, 7460.57) | 6790.1 (6459.14, 7138.02) | 6961.86 (6622.52, 7318.58) | 7096.92 (6751, 7460.57) | 4413.88 (4283.87, 4547.84) | 4466.91 (4335.34, 4602.47) | 4434.91 (4304.28, 4569.5) | 4410.44 (4280.54, 4544.29) | 5568.39 (5431.31, 5708.94) | 5422.4 (5288.91, 5559.26) | 5491.18 (5355.99, 5629.77) | 5544.67 (5408.17, 5684.62) |
| 2028 | 7148.81 (6798.45, 7517.21) | 6839.74 (6504.54, 7192.22) | 6999.29 (6656.26, 7359.99) | 7148.81 (6798.45, 7517.21) | 4401.68 (4264.33, 4543.45) | 4454.56 (4315.57, 4598.04) | 4425.1 (4287.02, 4567.62) | 4398.25 (4261.01, 4539.92) | 5509.38 (5372.37, 5649.88) | 5362.87 (5229.5, 5499.63) | 5425.62 (5290.7, 5563.99) | 5483.8 (5347.42, 5623.64) |
| 2029 | 7200.2 (6845.89, 7572.84) | 6888.91 (6549.92, 7245.44) | 7036.07 (6689.84, 7400.22) | 7200.2 (6845.89, 7572.84) | 4382.37 (4244, 4525.24) | 4435.02 (4294.99, 4579.61) | 4408.11 (4268.93, 4551.83) | 4378.95 (4240.69, 4521.72) | 5762.17 (5601.08, 5927.89) | 5606.78 (5450.04, 5768.03) | 5666.89 (5508.47, 5829.87) | 5733.21 (5572.93, 5898.09) |
| 2030 | 7251.2 (6893.3, 7627.68) | 6937.71 (6595.28, 7297.91) | 7072.31 (6723.24, 7439.5) | 7251.2 (6893.3, 7627.68) | 4387.54 (4250.7, 4528.79) | 4440.26 (4301.77, 4583.2) | 4415.76 (4278.04, 4557.92) | 4384.13 (4247.39, 4525.26) | 5745.35 (5587.01, 5908.17) | 5588.26 (5434.25, 5746.63) | 5642.7 (5487.19, 5802.62) | 5714.27 (5556.79, 5876.21) |
| 2031 | 7301.9 (6940.68, 7681.91) | 6986.22 (6640.61, 7349.8) | 7108.08 (6756.45, 7478.01) | 7301.9 (6940.68, 7681.91) | 4428.32 (4287.42, 4573.85) | 4481.52 (4338.93, 4628.8) | 4459.27 (4317.38, 4605.82) | 4424.87 (4284.08, 4570.29) | 5769.27 (5612.82, 5930.07) | 5609.36 (5457.25, 5765.71) | 5658.52 (5505.08, 5816.24) | 5735.85 (5580.31, 5895.72) |
| 2032 | 7352.37 (6988.04, 7735.7) | 7034.51 (6685.93, 7401.26) | 7143.48 (6789.5, 7515.92) | 7352.37 (6988.04, 7735.7) | 4491.54 (4345.17, 4642.83) | 4545.5 (4397.37, 4698.61) | 4525.43 (4377.96, 4677.87) | 4488.04 (4341.78, 4639.22) | 5832.8 (5666.84, 6003.62) | 5668.95 (5507.65, 5834.97) | 5713.09 (5550.53, 5880.41) | 5796.78 (5631.84, 5966.55) |
| 2033 | 7402.69 (7035.4, 7789.16) | 7082.65 (6731.24, 7452.41) | 7178.56 (6822.39, 7553.33) | 7402.69 (7035.4, 7789.16) | 4551.24 (4403.32, 4704.12) | 4605.92 (4456.22, 4760.64) | 4588.12 (4439, 4742.24) | 4547.69 (4399.89, 4700.46) | 5810.41 (5644.03, 5981.7) | 5645.02 (5483.37, 5811.43) | 5683.46 (5520.71, 5851.01) | 5772.31 (5607.01, 5942.48) |
| 2034 | 7452.91 (7082.78, 7842.39) | 7130.7 (6776.57, 7503.34) | 7213.39 (6855.15, 7590.35) | 7452.91 (7082.78, 7842.39) | 4587.11 (4438.27, 4740.93) | 4642.22 (4491.59, 4797.89) | 4626.84 (4476.71, 4781.99) | 4583.53 (4434.81, 4737.24) | 5789.47 (5617.53, 5966.66) | 5622.5 (5455.53, 5794.59) | 5655.3 (5487.35, 5828.39) | 5749.28 (5578.54, 5925.25) |
| 2035 | 7503.09 (7130.2, 7895.49) | 7178.71 (6821.94, 7554.14) | 7248.01 (6887.79, 7627.07) | 7503.09 (7130.2, 7895.49) | 4597.47 (4446.09, 4753.99) | 4652.7 (4499.51, 4811.11) | 4639.85 (4487.08, 4797.82) | 4593.89 (4442.63, 4750.29) | 5815.03 (5645.18, 5989.98) | 5645.15 (5480.27, 5814.99) | 5672.58 (5506.9, 5843.25) | 5772.44 (5603.84, 5946.12) |
| 2036 | 7553.27 (7177.68, 7948.51) | 7226.72 (6867.37, 7604.87) | 7282.48 (6920.35, 7663.55) | 7553.27 (7177.68, 7948.51) | 4597.98 (4445.44, 4755.76) | 4653.22 (4498.85, 4812.9) | 4642.94 (4488.91, 4802.26) | 4594.4 (4441.98, 4752.06) | 5913.55 (5732.01, 6100.85) | 5738.59 (5562.41, 5920.34) | 5760.88 (5584.02, 5943.34) | 5867.99 (5687.84, 6053.84) |
| 2037 | 7603.49 (7225.25, 8001.53) | 7274.77 (6912.88, 7655.6) | 7316.83 (6952.85, 7699.86) | 7603.49 (7225.25, 8001.53) | 4608.59 (4456.26, 4766.14) | 4663.96 (4509.8, 4823.4) | 4656.23 (4502.32, 4815.4) | 4605 (4452.79, 4762.42) | 5796.35 (5616.9, 5981.52) | 5622.68 (5448.62, 5802.31) | 5639.06 (5464.49, 5819.21) | 5749.47 (5571.48, 5933.15) |
| 2038 | 7653.79 (7272.93, 8054.59) | 7322.89 (6958.5, 7706.37) | 7351.09 (6985.29, 7736.04) | 7653.79 (7272.93, 8054.59) | 4639.85 (4486.04, 4798.93) | 4695.59 (4539.93, 4856.59) | 4690.4 (4534.91, 4851.22) | 4636.23 (4482.54, 4795.2) | 5911.84 (5728.69, 6100.83) | 5732.5 (5554.92, 5915.77) | 5743.63 (5565.7, 5927.25) | 5861.77 (5680.17, 6049.16) |
| 2039 | 7704.2 (7320.74, 8107.73) | 7371.12 (7004.25, 7757.21) | 7385.3 (7017.72, 7772.13) | 7704.2 (7320.74, 8107.73) | 4687.56 (4530.9, 4849.64) | 4743.88 (4585.34, 4907.9) | 4741.26 (4582.8, 4905.19) | 4683.91 (4527.37, 4845.86) | 5962.08 (5776.59, 6153.51) | 5778.99 (5599.21, 5964.55) | 5784.6 (5604.64, 5970.34) | 5909.31 (5725.46, 6099.05) |
| 2040 | 7754.74 (7368.71, 8161) | 7419.48 (7050.14, 7808.17) | 7419.48 (7050.14, 7808.17) | 7754.74 (7368.71, 8161) | 4737.51 (4579.01, 4901.49) | 4794.43 (4634.03, 4960.38) | 4794.43 (4634.03, 4960.38) | 4733.82 (4575.45, 4897.68) | 5933.39 (5746.36, 6126.51) | 5748.98 (5567.76, 5936.09) | 5748.98 (5567.76, 5936.09) | 5878.61 (5693.31, 6069.95) |

**Supplementary table 4: Observed and projected all ages DALYs rate per 100,000 (95% PIs) for cardiovascular diseases for reference forecast and three alternative scenarios, 1990–2040: male, female and both sexes combined**

|  | Cardiovascular diseases | | | | | | | | | | | | |
| --- | --- | --- | --- | --- | --- | --- | --- | --- | --- | --- | --- | --- | --- |
|  | Male | | | | Female | | | | Both sexed combined | | | | |
| Year | Reference | Scenario 1 | Scenario 2 | Scenario 3 | Reference | Scenario 1 | Scenario 2 | Scenario 3 | Reference | Scenario 1 | Scenario 2 | Scenario 3 |  |
| 1990 | 4649.62 | 4649.62 | 4649.62 | 4649.62 | 3674.78 | 3674.78 | 3674.78 | 3674.78 | 4154.07 | 4154.07 | 4154.07 | 4154.07 |  |
| 1991 | 4640.73 | 4640.73 | 4640.73 | 4640.73 | 3668.23 | 3668.23 | 3668.23 | 3668.23 | 4146.27 | 4146.27 | 4146.27 | 4146.27 |  |
| 1992 | 4663.64 | 4663.64 | 4663.64 | 4663.64 | 3662.89 | 3662.89 | 3662.89 | 3662.89 | 4154.71 | 4154.71 | 4154.71 | 4154.71 |  |
| 1993 | 4655.25 | 4655.25 | 4655.25 | 4655.25 | 3635.19 | 3635.19 | 3635.19 | 3635.19 | 4136.37 | 4136.37 | 4136.37 | 4136.37 |  |
| 1994 | 4553.49 | 4553.49 | 4553.49 | 4553.49 | 3538.43 | 3538.43 | 3538.43 | 3538.43 | 4037.02 | 4037.02 | 4037.02 | 4037.02 |  |
| 1995 | 4625.99 | 4625.99 | 4625.99 | 4625.99 | 3573.72 | 3573.72 | 3573.72 | 3573.72 | 4090.44 | 4090.44 | 4090.44 | 4090.44 |  |
| 1996 | 4517.41 | 4517.41 | 4517.41 | 4517.41 | 3461.52 | 3461.52 | 3461.52 | 3461.52 | 3979.85 | 3979.85 | 3979.85 | 3979.85 |  |
| 1997 | 4463.75 | 4463.75 | 4463.75 | 4463.75 | 3404.34 | 3404.34 | 3404.34 | 3404.34 | 3924.21 | 3924.21 | 3924.21 | 3924.21 |  |
| 1998 | 4574.58 | 4574.58 | 4574.58 | 4574.58 | 3420.04 | 3420.04 | 3420.04 | 3420.04 | 3986.36 | 3986.36 | 3986.36 | 3986.36 |  |
| 1999 | 4624.80 | 4624.80 | 4624.80 | 4624.80 | 3420.55 | 3420.55 | 3420.55 | 3420.55 | 4010.97 | 4010.97 | 4010.97 | 4010.97 |  |
| 2000 | 4523.51 | 4523.51 | 4523.51 | 4523.51 | 3344.29 | 3344.29 | 3344.29 | 3344.29 | 3922.16 | 3922.16 | 3922.16 | 3922.16 |  |
| 2001 | 4512.85 | 4512.85 | 4512.85 | 4512.85 | 3295.81 | 3295.81 | 3295.81 | 3295.81 | 3891.93 | 3891.93 | 3891.93 | 3891.93 |  |
| 2002 | 4489.96 | 4489.96 | 4489.96 | 4489.96 | 3260.85 | 3260.85 | 3260.85 | 3260.85 | 3862.57 | 3862.57 | 3862.57 | 3862.57 |  |
| 2003 | 4582.90 | 4582.90 | 4582.90 | 4582.90 | 3258.75 | 3258.75 | 3258.75 | 3258.75 | 3906.65 | 3906.65 | 3906.65 | 3906.65 |  |
| 2004 | 4536.52 | 4536.52 | 4536.52 | 4536.52 | 3286.23 | 3286.23 | 3286.23 | 3286.23 | 3897.66 | 3897.66 | 3897.66 | 3897.66 |  |
| 2005 | 4646.18 | 4646.18 | 4646.18 | 4646.18 | 3313.56 | 3313.56 | 3313.56 | 3313.56 | 3964.90 | 3964.90 | 3964.90 | 3964.90 |  |
| 2006 | 4581.96 | 4581.96 | 4581.96 | 4581.96 | 3291.39 | 3291.39 | 3291.39 | 3291.39 | 3921.84 | 3921.84 | 3921.84 | 3921.84 |  |
| 2007 | 4605.24 | 4605.24 | 4605.24 | 4605.24 | 3287.08 | 3287.08 | 3287.08 | 3287.08 | 3930.70 | 3930.70 | 3930.70 | 3930.70 |  |
| 2008 | 4609.49 | 4609.49 | 4609.49 | 4609.49 | 3293.43 | 3293.43 | 3293.43 | 3293.43 | 3935.75 | 3935.75 | 3935.75 | 3935.75 |  |
| 2009 | 4630.87 | 4630.87 | 4630.87 | 4630.87 | 3256.07 | 3256.07 | 3256.07 | 3256.07 | 3926.79 | 3926.79 | 3926.79 | 3926.79 |  |
| 2010 | 4664.06 | 4664.06 | 4664.06 | 4664.06 | 3300.70 | 3300.70 | 3300.70 | 3300.70 | 3965.59 | 3965.59 | 3965.59 | 3965.59 |  |
| 2011 | 4686.29 | 4686.29 | 4686.29 | 4686.29 | 3389.84 | 3389.84 | 3389.84 | 3389.84 | 4021.94 | 4021.94 | 4021.94 | 4021.94 |  |
| 2012 | 4648.77 | 4648.77 | 4648.77 | 4648.77 | 3357.85 | 3357.85 | 3357.85 | 3357.85 | 3987.16 | 3987.16 | 3987.16 | 3987.16 |  |
| 2013 | 4618.32 | 4618.32 | 4618.32 | 4618.32 | 3363.72 | 3363.72 | 3363.72 | 3363.72 | 3975.24 | 3975.24 | 3975.24 | 3975.24 |  |
| 2014 | 4565.68 | 4565.68 | 4565.68 | 4565.68 | 3373.60 | 3373.60 | 3373.60 | 3373.60 | 3954.60 | 3954.60 | 3954.60 | 3954.60 |  |
| 2015 | 4519.85 | 4519.85 | 4519.85 | 4519.85 | 3340.20 | 3340.20 | 3340.20 | 3340.20 | 3915.10 | 3915.10 | 3915.10 | 3915.10 |  |
| 2016 | 4590.29 | 4590.29 | 4590.29 | 4590.29 | 3400.76 | 3400.76 | 3400.76 | 3400.76 | 3980.42 | 3980.42 | 3980.42 | 3980.42 |  |
| 2017 | 4598.70 (4495.52, 4704.26) | 4598.70 (4495.52, 4704.26) | 4598.70 (4495.52, 4704.26) | 4598.70 (4495.52, 4704.26) | 3256.37 (3186.71, 3327.55) | 3251.38 (3181.83, 3322.45) | 3251.38 (3181.83, 3322.45) | 3251.38 (3181.83, 3322.45) | 4038.39 (3967.17, 4110.89) | 4029.63 (3958.56, 4101.96) | 4029.63 (3958.56, 4101.96) | 4029.63 (3958.56, 4101.96) |  |
| 2018 | 4597.59 (4494.43, 4703.12) | 4592.74 (4489.69, 4698.16) | 4596.32 (4493.19, 4701.82) | 4597.59 (4494.43, 4703.12) | 3386.8 (3264.69, 3513.49) | 3396.84 (3274.36, 3523.9) | 3384.77 (3262.73, 3511.38) | 3380.52 (3258.63, 3506.97) | 4002.37 (3863.25, 4146.5) | 3909.32 (3773.44, 4050.11) | 3965.62 (3827.78, 4108.43) | 3985.68 (3847.14, 4129.22) |  |
| 2019 | 4595.73 (4492.61, 4701.22) | 4586.05 (4483.15, 4691.31) | 4593.2 (4490.14, 4698.63) | 4595.73 (4492.61, 4701.22) | 3354.66 (3228.83, 3485.38) | 3381.24 (3254.42, 3513) | 3357.25 (3231.34, 3488.08) | 3348.83 (3223.23, 3479.33) | 4009.63 (3869.02, 4155.35) | 3833.69 (3699.25, 3973.02) | 3944.9 (3806.56, 4088.27) | 3984.92 (3845.17, 4129.74) |  |
| 2020 | 4594.14 (4491.05, 4699.59) | 4579.63 (4476.87, 4684.75) | 4590.35 (4487.35, 4695.71) | 4594.14 (4491.05, 4699.59) | 3370.11 (3232.88, 3513.16) | 3413.06 (3274.09, 3557.94) | 3376.82 (3239.31, 3520.15) | 3364.11 (3227.13, 3506.91) | 3960.8 (3818.97, 4107.91) | 3707.02 (3574.27, 3844.7) | 3869.48 (3730.92, 4013.2) | 3928.51 (3787.83, 4074.41) |  |
| 2021 | 4592.45 (4489.41, 4697.86) | 4573.12 (4470.51, 4678.09) | 4587.4 (4484.47, 4692.7) | 4592.45 (4489.41, 4697.86) | 3383.63 (3239.31, 3534.39) | 3443.35 (3296.48, 3596.77) | 3394.68 (3249.88, 3545.93) | 3377.66 (3233.59, 3528.15) | 3975.32 (3832.9, 4123.04) | 3642.02 (3511.54, 3777.35) | 3856.38 (3718.22, 3999.68) | 3935.02 (3794.04, 4081.23) |  |
| 2022 | 4590.8 (4487.79, 4696.17) | 4566.66 (4464.19, 4671.48) | 4584.49 (4481.62, 4689.72) | 4590.8 (4487.79, 4696.17) | 3259.21 (3118.21, 3406.59) | 3332.72 (3188.54, 3483.43) | 3273.94 (3132.3, 3421.99) | 3253.44 (3112.69, 3400.56) | 4008.26 (3861.03, 4161.1) | 3594.63 (3462.59, 3731.7) | 3861.01 (3719.19, 4008.24) | 3959.67 (3814.23, 4110.66) |  |
| 2023 | 4589.14 (4486.17, 4694.47) | 4560.2 (4457.87, 4664.87) | 4581.57 (4478.77, 4686.73) | 4589.14 (4486.17, 4694.47) | 3296.27 (3152.83, 3446.23) | 3386.89 (3239.51, 3540.98) | 3315.34 (3171.07, 3466.17) | 3290.44 (3147.26, 3440.14) | 4015.05 (3863.78, 4172.24) | 3524.66 (3391.87, 3662.65) | 3840.38 (3695.69, 3990.73) | 3958.44 (3809.3, 4113.41) |  |
| 2024 | 4587.48 (4484.55, 4692.78) | 4558.55 (4456.26, 4663.18) | 4578.66 (4475.92, 4683.75) | 4587.48 (4484.55, 4692.78) | 3198.04 (3056.51, 3346.13) | 3285.97 (3140.54, 3438.12) | 3220.58 (3078.05, 3369.71) | 3192.39 (3051.11, 3340.21) | 4045.68 (3891.68, 4205.77) | 3544.44 (3409.52, 3684.7) | 3842.49 (3696.23, 3994.54) | 3980.65 (3829.12, 4138.17) |  |
| 2025 | 4585.82 (4482.92, 4691.08) | 4556.9 (4454.65, 4661.5) | 4575.74 (4473.07, 4680.77) | 4585.82 (4482.92, 4691.08) | 3258.02 (3104.94, 3418.64) | 3347.59 (3190.3, 3512.63) | 3285.1 (3130.75, 3447.07) | 3252.26 (3099.45, 3412.6) | 4010.31 (3857.78, 4168.87) | 3506.41 (3373.05, 3645.05) | 3782.13 (3638.28, 3931.67) | 3937.94 (3788.16, 4093.64) |  |
| 2026 | 4584.16 (4481.3, 4689.38) | 4555.25 (4453.04, 4659.81) | 4572.83 (4470.22, 4677.79) | 4584.16 (4481.3, 4689.38) | 3252.59 (3098.02, 3414.88) | 3342.02 (3183.2, 3508.76) | 3283.76 (3127.71, 3447.6) | 3246.84 (3092.55, 3408.84) | 3989.66 (3837.78, 4147.54) | 3481.37 (3348.84, 3619.14) | 3736.22 (3593.99, 3884.07) | 3909.82 (3760.98, 4064.54) |  |
| 2027 | 4582.51 (4479.68, 4687.69) | 4553.61 (4451.43, 4658.13) | 4569.92 (4467.38, 4674.81) | 4582.51 (4479.68, 4687.69) | 3263.96 (3106.72, 3429.16) | 3353.69 (3192.13, 3523.44) | 3299.37 (3140.43, 3466.37) | 3258.19 (3101.22, 3423.1) | 3996.38 (3838.33, 4160.94) | 3480.25 (3342.61, 3623.56) | 3716.22 (3569.25, 3869.25) | 3908.56 (3753.98, 4069.51) |  |
| 2028 | 4580.85 (4478.06, 4686) | 4551.96 (4449.82, 4656.44) | 4567.01 (4464.54, 4671.84) | 4580.85 (4478.06, 4686) | 3285.75 (3127.3, 3452.23) | 3376.08 (3213.28, 3547.14) | 3325.57 (3165.2, 3494.07) | 3279.94 (3121.77, 3446.13) | 3994.76 (3837.12, 4158.88) | 3471.88 (3334.87, 3614.51) | 3688.61 (3543.05, 3840.15) | 3899.15 (3745.29, 4059.34) |  |
| 2029 | 4579.19 (4476.45, 4684.3) | 4550.32 (4448.22, 4654.76) | 4564.1 (4461.69, 4668.86) | 4579.19 (4476.45, 4684.3) | 3213.07 (3057.57, 3376.48) | 3301.41 (3141.64, 3469.31) | 3256.11 (3098.52, 3421.7) | 3207.39 (3052.17, 3370.51) | 4039.72 (3879.77, 4206.28) | 3503.92 (3365.18, 3648.38) | 3703.92 (3557.26, 3856.63) | 3935.15 (3779.33, 4097.39) |  |
| 2030 | 4577.54 (4474.83, 4682.61) | 4548.67 (4446.61, 4653.08) | 4561.2 (4458.85, 4665.89) | 4577.54 (4474.83, 4682.61) | 3213.81 (3058.18, 3377.36) | 3302.17 (3142.26, 3470.21) | 3260.95 (3103.04, 3426.89) | 3208.13 (3052.77, 3371.39) | 4043.76 (3883.53, 4210.6) | 3500.4 (3361.7, 3644.82) | 3681.57 (3535.69, 3833.47) | 3931.19 (3775.42, 4093.38) |  |
| 2031 | 4575.88 (4473.21, 4680.92) | 4547.03 (4445, 4651.4) | 4558.3 (4456.02, 4662.92) | 4575.88 (4473.21, 4680.92) | 3138.1 (2983.94, 3300.23) | 3224.37 (3065.97, 3390.96) | 3188.13 (3031.51, 3352.84) | 3132.55 (2978.66, 3294.39) | 4029.3 (3866.43, 4199.02) | 3480.89 (3340.2, 3627.52) | 3642.63 (3495.39, 3796.07) | 3909.28 (3751.27, 4073.95) |  |
| 2032 | 4574.23 (4471.59, 4679.22) | 4545.38 (4443.39, 4649.71) | 4555.4 (4453.18, 4659.96) | 4574.23 (4471.59, 4679.22) | 3146.63 (2990.64, 3310.77) | 3233.14 (3072.86, 3401.79) | 3200.82 (3042.13, 3367.77) | 3141.07 (2985.35, 3304.91) | 4022.51 (3859.22, 4192.71) | 3468.08 (3327.29, 3614.81) | 3610.95 (3464.36, 3763.73) | 3894.89 (3736.78, 4059.68) |  |
| 2033 | 4572.58 (4469.98, 4677.53) | 4543.74 (4441.79, 4648.03) | 4552.5 (4450.35, 4656.99) | 4572.58 (4469.98, 4677.53) | 3145.64 (2989.58, 3309.85) | 3232.12 (3071.77, 3400.85) | 3203.83 (3044.88, 3371.07) | 3140.08 (2984.29, 3304) | 3994.94 (3831.81, 4165.01) | 3437.4 (3297.04, 3583.74) | 3560.99 (3415.59, 3712.59) | 3860.44 (3702.8, 4024.78) |  |
| 2034 | 4570.92 (4468.36, 4675.84) | 4542.1 (4440.18, 4646.35) | 4549.6 (4447.52, 4654.03) | 4570.92 (4468.36, 4675.84) | 3158.12 (2999.97, 3324.62) | 3244.95 (3082.45, 3416.02) | 3220.59 (3059.3, 3390.37) | 3152.54 (2994.67, 3318.74) | 4017.32 (3853.23, 4188.39) | 3449.74 (3308.83, 3596.64) | 3555.78 (3410.55, 3707.2) | 3874.29 (3716.05, 4039.27) |  |
| 2035 | 4569.27 (4466.75, 4674.15) | 4540.46 (4438.58, 4644.67) | 4546.7 (4444.68, 4651.07) | 4569.27 (4466.75, 4674.15) | 3185.9 (3026.5, 3353.71) | 3273.49 (3109.71, 3445.91) | 3253 (3090.24, 3424.34) | 3180.27 (3021.15, 3347.78) | 4038.83 (3873.54, 4211.18) | 3461.27 (3319.61, 3608.97) | 3549.71 (3404.44, 3701.19) | 3887.24 (3728.15, 4053.12) |  |
| 2036 | 4567.62 (4465.13, 4672.46) | 4538.81 (4436.97, 4642.99) | 4543.81 (4441.86, 4648.11) | 4567.62 (4465.13, 4672.46) | 3149.61 (2991.78, 3315.77) | 3236.2 (3074.03, 3406.93) | 3219.98 (3058.63, 3389.85) | 3144.04 (2986.49, 3309.91) | 4047.44 (3878.98, 4223.22) | 3461.7 (3317.61, 3612.04) | 3532.28 (3385.26, 3685.69) | 3887.72 (3725.91, 4056.57) |  |
| 2037 | 4565.97 (4463.52, 4670.77) | 4537.17 (4435.37, 4641.32) | 4540.92 (4439.03, 4645.15) | 4565.97 (4463.52, 4670.77) | 3138.57 (2981.26, 3304.18) | 3224.86 (3063.22, 3395.02) | 3212.73 (3051.7, 3382.25) | 3133.02 (2975.99, 3298.34) | 4058.56 (3889.47, 4235) | 3464.26 (3319.93, 3614.86) | 3517.1 (3370.57, 3670) | 3890.6 (3728.51, 4059.74) |  |
| 2038 | 4564.32 (4461.9, 4669.08) | 4535.53 (4433.76, 4639.64) | 4538.03 (4436.2, 4642.19) | 4564.32 (4461.9, 4669.08) | 3078.63 (2922.97, 3242.59) | 3163.27 (3003.33, 3331.74) | 3155.34 (2995.79, 3323.38) | 3073.19 (2917.8, 3236.86) | 4028.72 (3860.97, 4203.75) | 3431.9 (3289, 3581) | 3466.71 (3322.36, 3617.33) | 3854.25 (3693.77, 4021.71) |  |
| 2039 | 4562.67 (4460.29, 4667.4) | 4533.89 (4432.16, 4637.96) | 4535.14 (4433.38, 4639.24) | 4562.67 (4460.29, 4667.4) | 3059.39 (2904.62, 3222.4) | 3143.5 (2984.48, 3311) | 3139.55 (2980.73, 3306.84) | 3053.98 (2899.49, 3216.71) | 4021.25 (3853.83, 4195.94) | 3418.67 (3276.34, 3567.19) | 3435.97 (3292.92, 3585.24) | 3839.4 (3679.55, 4006.2) |  |
| 2040 | 4561.02 (4458.68, 4665.71) | 4532.25 (4430.56, 4636.28) | 4532.25 (4430.56, 4636.28) | 4561.02 (4458.68, 4665.71) | 3049.23 (2894.99, 3211.69) | 3133.06 (2974.58, 3299.99) | 3133.06 (2974.58, 3299.99) | 3043.84 (2889.87, 3206.01) | 4027.69 (3857.54, 4205.35) | 3417.29 (3272.93, 3568.02) | 3417.29 (3272.93, 3568.02) | 3837.85 (3675.73, 4007.14) |  |

**Supplementary table 5: Observed and projected all ages DALYs rate per 100,000 (95% PIs) for diabetes and kidney disease for reference forecast and three alternative scenarios, 1990–2040: male, female and both sexes combined**

|  | Diabetes and kidney diseases | | | | | | | | | | | | |
| --- | --- | --- | --- | --- | --- | --- | --- | --- | --- | --- | --- | --- | --- |
|  | Male | | | | Female | | | | Both sexed combined | | | | |
| Year | Reference | Scenario 1 | Scenario 2 | Scenario 3 | Reference | Scenario 1 | Scenario 2 | Scenario 3 | Reference | Scenario 1 | Scenario 2 | Scenario 3 |  |
| 1990 | 950.84 | 950.84 | 950.84 | 950.84 | 828.05 | 828.05 | 828.05 | 828.05 | 888.43 | 888.43 | 888.43 | 888.43 |  |
| 1991 | 963.79 | 963.79 | 963.79 | 963.79 | 832.44 | 832.44 | 832.44 | 832.44 | 897.00 | 897.00 | 897.00 | 897.00 |  |
| 1992 | 980.63 | 980.63 | 980.63 | 980.63 | 837.75 | 837.75 | 837.75 | 837.75 | 907.97 | 907.97 | 907.97 | 907.97 |  |
| 1993 | 993.72 | 993.72 | 993.72 | 993.72 | 838.69 | 838.69 | 838.69 | 838.69 | 914.86 | 914.86 | 914.86 | 914.86 |  |
| 1994 | 995.42 | 995.42 | 995.42 | 995.42 | 831.50 | 831.50 | 831.50 | 831.50 | 912.01 | 912.01 | 912.01 | 912.01 |  |
| 1995 | 1014.66 | 1014.66 | 1014.66 | 1014.66 | 839.43 | 839.43 | 839.43 | 839.43 | 925.48 | 925.48 | 925.48 | 925.48 |  |
| 1996 | 1010.01 | 1010.01 | 1010.01 | 1010.01 | 829.32 | 829.32 | 829.32 | 829.32 | 918.02 | 918.02 | 918.02 | 918.02 |  |
| 1997 | 1015.69 | 1015.69 | 1015.69 | 1015.69 | 829.61 | 829.61 | 829.61 | 829.61 | 920.92 | 920.92 | 920.92 | 920.92 |  |
| 1998 | 1040.29 | 1040.29 | 1040.29 | 1040.29 | 839.31 | 839.31 | 839.31 | 839.31 | 937.89 | 937.89 | 937.89 | 937.89 |  |
| 1999 | 1058.08 | 1058.08 | 1058.08 | 1058.08 | 847.41 | 847.41 | 847.41 | 847.41 | 950.70 | 950.70 | 950.70 | 950.70 |  |
| 2000 | 1058.05 | 1058.05 | 1058.05 | 1058.05 | 844.29 | 844.29 | 844.29 | 844.29 | 949.04 | 949.04 | 949.04 | 949.04 |  |
| 2001 | 1059.48 | 1059.48 | 1059.48 | 1059.48 | 840.02 | 840.02 | 840.02 | 840.02 | 947.51 | 947.51 | 947.51 | 947.51 |  |
| 2002 | 1060.22 | 1060.22 | 1060.22 | 1060.22 | 833.90 | 833.90 | 833.90 | 833.90 | 944.70 | 944.70 | 944.70 | 944.70 |  |
| 2003 | 1073.79 | 1073.79 | 1073.79 | 1073.79 | 830.99 | 830.99 | 830.99 | 830.99 | 949.79 | 949.79 | 949.79 | 949.79 |  |
| 2004 | 1075.25 | 1075.25 | 1075.25 | 1075.25 | 833.79 | 833.79 | 833.79 | 833.79 | 951.87 | 951.87 | 951.87 | 951.87 |  |
| 2005 | 1097.64 | 1097.64 | 1097.64 | 1097.64 | 841.73 | 841.73 | 841.73 | 841.73 | 966.81 | 966.81 | 966.81 | 966.81 |  |
| 2006 | 1110.53 | 1110.53 | 1110.53 | 1110.53 | 852.41 | 852.41 | 852.41 | 852.41 | 978.50 | 978.50 | 978.50 | 978.50 |  |
| 2007 | 1140.91 | 1140.91 | 1140.91 | 1140.91 | 871.35 | 871.35 | 871.35 | 871.35 | 1002.97 | 1002.97 | 1002.97 | 1002.97 |  |
| 2008 | 1171.85 | 1171.85 | 1171.85 | 1171.85 | 894.55 | 894.55 | 894.55 | 894.55 | 1029.89 | 1029.89 | 1029.89 | 1029.89 |  |
| 2009 | 1203.12 | 1203.12 | 1203.12 | 1203.12 | 911.12 | 911.12 | 911.12 | 911.12 | 1053.58 | 1053.58 | 1053.58 | 1053.58 |  |
| 2010 | 1228.51 | 1228.51 | 1228.51 | 1228.51 | 934.34 | 934.34 | 934.34 | 934.34 | 1077.80 | 1077.80 | 1077.80 | 1077.80 |  |
| 2011 | 1235.38 | 1235.38 | 1235.38 | 1235.38 | 956.44 | 956.44 | 956.44 | 956.44 | 1092.44 | 1092.44 | 1092.44 | 1092.44 |  |
| 2012 | 1236.30 | 1236.30 | 1236.30 | 1236.30 | 964.31 | 964.31 | 964.31 | 964.31 | 1096.90 | 1096.90 | 1096.90 | 1096.90 |  |
| 2013 | 1237.02 | 1237.02 | 1237.02 | 1237.02 | 975.38 | 975.38 | 975.38 | 975.38 | 1102.91 | 1102.91 | 1102.91 | 1102.91 |  |
| 2014 | 1234.63 | 1234.63 | 1234.63 | 1234.63 | 986.83 | 986.83 | 986.83 | 986.83 | 1107.60 | 1107.60 | 1107.60 | 1107.60 |  |
| 2015 | 1230.98 | 1230.98 | 1230.98 | 1230.98 | 990.17 | 990.17 | 990.17 | 990.17 | 1107.53 | 1107.53 | 1107.53 | 1107.53 |  |
| 2016 | 1235.05 | 1235.05 | 1235.05 | 1235.05 | 1003.63 | 1003.63 | 1003.63 | 1003.63 | 1116.40 | 1116.40 | 1116.40 | 1116.40 |  |
| 2017 | 1238.35 (1215.1, 1262.04) | 1238.35 (1215.1, 1262.04) | 1238.35 (1215.1, 1262.04) | 1238.35 (1215.1, 1262.04) | 1011.1 (993.47, 1029.05) | 1010.57 (992.94, 1028.51) | 1010.57 (992.94, 1028.51) | 1010.57 (992.94, 1028.51) | 1118.04 (1098.81, 1137.61) | 1118.08 (1098.85, 1137.65) | 1118.08 (1098.85, 1137.65) | 1118.08 (1098.85, 1137.65) |  |
| 2018 | 1242.97 (1206.32, 1280.72) | 1240.24 (1203.67, 1277.91) | 1242.25 (1205.63, 1279.99) | 1242.97 (1206.32, 1280.72) | 1014.54 (980.55, 1049.72) | 1015.58 (981.55, 1050.79) | 1014.33 (980.34, 1049.5) | 1013.89 (979.92, 1049.04) | 1119.54 (1084.53, 1155.67) | 1119.98 (1084.95, 1156.13) | 1119.71 (1084.7, 1155.85) | 1119.62 (1084.61, 1155.76) |  |
| 2019 | 1249.97 (1200.84, 1301.12) | 1244.49 (1195.57, 1295.41) | 1248.54 (1199.46, 1299.63) | 1249.97 (1200.84, 1301.12) | 1015.4 (966.34, 1066.94) | 1018.17 (968.98, 1069.86) | 1015.67 (966.6, 1067.23) | 1014.79 (965.76, 1066.3) | 1121.27 (1073.22, 1171.47) | 1122.11 (1074.03, 1172.35) | 1121.58 (1073.52, 1171.79) | 1121.39 (1073.33, 1171.59) |  |
| 2020 | 1258.68 (1200.71, 1319.45) | 1250.41 (1192.82, 1310.78) | 1256.52 (1198.65, 1317.18) | 1258.68 (1200.71, 1319.45) | 1014.38 (952.5, 1080.28) | 1018.82 (956.68, 1085.01) | 1015.08 (953.16, 1081.02) | 1013.75 (951.92, 1079.61) | 1123.08 (1065.09, 1184.23) | 1124.32 (1066.27, 1185.54) | 1123.52 (1065.5, 1184.69) | 1123.24 (1065.24, 1184.39) |  |
| 2021 | 1269.05 (1205.27, 1336.2) | 1257.94 (1194.72, 1324.51) | 1266.14 (1202.51, 1333.14) | 1269.05 (1205.27, 1336.2) | 1012.4 (940.19, 1090.16) | 1018.54 (945.88, 1096.77) | 1013.54 (941.24, 1091.39) | 1011.78 (939.61, 1089.5) | 1125.51 (1060.36, 1194.66) | 1127.15 (1061.91, 1196.4) | 1126.08 (1060.9, 1195.26) | 1125.7 (1060.54, 1194.86) |  |
| 2022 | 1280.63 (1213.62, 1351.33) | 1266.63 (1200.36, 1336.57) | 1276.96 (1210.15, 1347.46) | 1280.63 (1213.62, 1351.33) | 1010.05 (929.86, 1097.15) | 1017.86 (937.05, 1105.63) | 1011.62 (931.31, 1098.86) | 1009.43 (929.29, 1096.48) | 1128.61 (1058.56, 1203.29) | 1130.66 (1060.48, 1205.48) | 1129.31 (1059.22, 1204.04) | 1128.84 (1058.78, 1203.54) |  |
| 2023 | 1293.02 (1224.44, 1365.44) | 1276.08 (1208.4, 1347.56) | 1288.58 (1220.23, 1360.75) | 1293.02 (1224.44, 1365.44) | 1007.79 (921.69, 1101.93) | 1017.28 (930.37, 1112.3) | 1009.8 (923.53, 1104.13) | 1007.18 (921.13, 1101.26) | 1132.45 (1059.17, 1210.8) | 1134.91 (1061.47, 1213.43) | 1133.29 (1059.96, 1211.69) | 1132.72 (1059.42, 1211.08) |  |
| 2024 | 1305.8 (1236.41, 1379.08) | 1288.7 (1220.22, 1361.02) | 1300.57 (1231.46, 1373.56) | 1305.8 (1236.41, 1379.08) | 1005.95 (915.64, 1105.18) | 1015.42 (924.25, 1115.58) | 1008.39 (917.86, 1107.86) | 1005.34 (915.08, 1104.5) | 1136.99 (1061.64, 1217.68) | 1139.49 (1063.98, 1220.36) | 1137.96 (1062.55, 1218.72) | 1137.29 (1061.93, 1218.01) |  |
| 2025 | 1318.62 (1248.53, 1392.64) | 1301.35 (1232.18, 1374.4) | 1312.59 (1242.82, 1386.27) | 1318.62 (1248.53, 1392.64) | 1004.73 (911.52, 1107.49) | 1014.19 (920.09, 1117.91) | 1007.61 (914.13, 1110.66) | 1004.12 (910.96, 1106.81) | 1142.15 (1065.48, 1224.33) | 1144.7 (1067.87, 1227.07) | 1143.26 (1066.52, 1225.53) | 1142.5 (1065.81, 1224.7) |  |
| 2026 | 1331.21 (1260.19, 1406.23) | 1313.77 (1243.68, 1387.81) | 1324.36 (1253.7, 1399) | 1331.21 (1260.19, 1406.23) | 1004.25 (909.1, 1109.36) | 1013.7 (917.65, 1119.8) | 1007.56 (912.1, 1113.02) | 1003.64 (908.54, 1108.68) | 1147.85 (1070.31, 1231.01) | 1150.45 (1072.74, 1233.8) | 1149.1 (1071.48, 1232.35) | 1148.23 (1070.67, 1231.42) |  |
| 2027 | 1343.4 (1271.2, 1419.7) | 1325.8 (1254.55, 1401.11) | 1335.72 (1263.93, 1411.59) | 1343.4 (1271.2, 1419.7) | 1004.54 (908.14, 1111.18) | 1014 (916.68, 1121.64) | 1008.29 (911.53, 1115.33) | 1003.93 (907.58, 1110.51) | 1153.99 (1075.81, 1237.84) | 1156.64 (1078.29, 1240.69) | 1155.38 (1077.11, 1239.34) | 1154.41 (1076.21, 1238.3) |  |
| 2028 | 1355.12 (1281.65, 1432.8) | 1337.37 (1264.86, 1414.03) | 1346.6 (1273.59, 1423.79) | 1355.12 (1281.65, 1432.8) | 1005.61 (908.39, 1113.23) | 1015.07 (916.94, 1123.7) | 1009.8 (912.17, 1117.87) | 1004.99 (907.83, 1112.55) | 1160.47 (1081.77, 1244.89) | 1163.18 (1084.3, 1247.8) | 1162.01 (1083.21, 1246.55) | 1160.94 (1082.21, 1245.4) |  |
| 2029 | 1366.38 (1291.73, 1445.35) | 1348.49 (1274.81, 1426.42) | 1357.02 (1282.88, 1435.44) | 1366.38 (1291.73, 1445.35) | 1007.39 (909.62, 1115.67) | 1016.87 (918.18, 1126.17) | 1012.03 (913.81, 1120.81) | 1006.78 (909.07, 1114.99) | 1167.22 (1088.04, 1252.17) | 1169.99 (1090.62, 1255.13) | 1168.91 (1089.61, 1253.98) | 1167.73 (1088.52, 1252.71) |  |
| 2030 | 1377.27 (1301.63, 1457.31) | 1359.23 (1284.58, 1438.22) | 1367.05 (1291.96, 1446.49) | 1377.27 (1301.63, 1457.31) | 1009.84 (911.65, 1118.59) | 1019.34 (920.23, 1129.12) | 1014.93 (916.25, 1124.23) | 1009.22 (911.1, 1117.91) | 1174.18 (1094.52, 1259.63) | 1177 (1097.15, 1262.66) | 1176.01 (1096.23, 1261.6) | 1174.73 (1095.03, 1260.22) |  |
| 2031 | 1387.9 (1311.46, 1468.79) | 1369.72 (1294.28, 1449.55) | 1376.8 (1300.98, 1457.05) | 1387.9 (1311.46, 1468.79) | 1012.86 (914.31, 1122.03) | 1022.39 (922.92, 1132.59) | 1018.41 (919.32, 1128.17) | 1012.24 (913.75, 1121.34) | 1181.27 (1101.14, 1267.24) | 1184.15 (1103.82, 1270.33) | 1183.26 (1102.99, 1269.37) | 1181.87 (1101.69, 1267.88) |  |
| 2032 | 1398.39 (1321.3, 1479.98) | 1380.07 (1303.99, 1460.59) | 1386.42 (1309.98, 1467.31) | 1398.39 (1321.3, 1479.98) | 1016.38 (917.47, 1125.95) | 1025.94 (926.1, 1136.54) | 1022.39 (922.89, 1132.6) | 1015.76 (916.91, 1125.26) | 1188.47 (1107.84, 1274.97) | 1191.41 (1110.58, 1278.12) | 1190.61 (1109.84, 1277.26) | 1189.11 (1108.44, 1275.65) |  |
| 2033 | 1408.88 (1331.2, 1491.09) | 1390.42 (1313.76, 1471.56) | 1396.02 (1319.05, 1477.48) | 1408.88 (1331.2, 1491.09) | 1020.3 (921.01, 1130.3) | 1029.91 (929.68, 1140.94) | 1026.78 (926.86, 1137.48) | 1019.68 (920.45, 1129.61) | 1195.74 (1114.62, 1282.77) | 1198.74 (1117.41, 1285.99) | 1198.03 (1116.75, 1285.23) | 1196.43 (1115.25, 1283.51) |  |
| 2034 | 1419.47 (1341.21, 1502.31) | 1400.88 (1323.64, 1482.63) | 1405.71 (1328.2, 1487.74) | 1419.47 (1341.21, 1502.31) | 1024.56 (924.86, 1135.02) | 1034.2 (933.56, 1145.7) | 1031.51 (931.13, 1142.72) | 1023.94 (924.29, 1134.32) | 1203.07 (1121.44, 1290.64) | 1206.12 (1124.29, 1293.91) | 1205.51 (1123.72, 1293.26) | 1203.79 (1122.12, 1291.42) |  |
| 2035 | 1430.26 (1351.38, 1513.74) | 1411.52 (1333.68, 1493.92) | 1415.58 (1337.5, 1498.21) | 1430.26 (1351.38, 1513.74) | 1029.08 (928.92, 1140.03) | 1038.76 (937.67, 1150.76) | 1036.51 (935.63, 1148.26) | 1028.45 (928.36, 1139.33) | 1210.43 (1128.3, 1298.54) | 1213.54 (1131.2, 1301.88) | 1213.03 (1130.72, 1301.33) | 1211.2 (1129.01, 1299.36) |  |
| 2036 | 1441.28 (1361.75, 1525.46) | 1422.4 (1343.91, 1505.48) | 1425.67 (1347, 1508.94) | 1441.28 (1361.75, 1525.46) | 1033.79 (933.16, 1145.26) | 1043.51 (941.94, 1156.04) | 1041.71 (940.31, 1154.03) | 1033.15 (932.59, 1144.56) | 1217.81 (1135.18, 1306.47) | 1220.99 (1138.13, 1309.87) | 1220.58 (1137.75, 1309.43) | 1218.63 (1135.94, 1307.34) |  |
| 2037 | 1452.56 (1372.36, 1537.46) | 1433.54 (1354.38, 1517.32) | 1436.01 (1356.71, 1519.94) | 1452.56 (1372.36, 1537.46) | 1038.64 (937.52, 1150.65) | 1048.41 (946.35, 1161.48) | 1047.05 (945.12, 1159.97) | 1038 (936.95, 1149.95) | 1225.23 (1142.08, 1314.42) | 1228.46 (1145.1, 1317.89) | 1228.15 (1144.81, 1317.56) | 1226.09 (1142.89, 1315.35) |  |
| 2038 | 1464.11 (1383.21, 1549.73) | 1444.93 (1365.09, 1529.43) | 1446.59 (1366.66, 1531.19) | 1464.11 (1383.21, 1549.73) | 1043.58 (941.97, 1156.15) | 1053.4 (950.83, 1167.03) | 1052.49 (950.01, 1166.02) | 1042.94 (941.39, 1155.44) | 1232.66 (1149.01, 1322.4) | 1235.96 (1152.08, 1325.94) | 1235.75 (1151.89, 1325.71) | 1233.57 (1149.86, 1323.38) |  |
| 2039 | 1475.88 (1394.3, 1562.23) | 1456.55 (1376.04, 1541.77) | 1457.38 (1376.83, 1542.65) | 1475.88 (1394.3, 1562.23) | 1048.58 (946.47, 1161.71) | 1058.45 (955.37, 1172.64) | 1057.99 (954.96, 1172.14) | 1047.94 (945.89, 1161) | 1240.13 (1155.97, 1330.41) | 1243.48 (1159.09, 1334.01) | 1243.38 (1159, 1333.9) | 1241.08 (1156.86, 1331.44) |  |
| 2040 | 1487.85 (1405.6, 1574.93) | 1468.37 (1387.19, 1554.3) | 1468.37 (1387.19, 1554.3) | 1487.85 (1405.6, 1574.93) | 1053.61 (950.99, 1167.31) | 1063.53 (959.94, 1178.29) | 1063.53 (959.94, 1178.29) | 1052.97 (950.41, 1166.59) | 1247.62 (1162.95, 1338.45) | 1251.03 (1166.13, 1342.11) | 1251.03 (1166.13, 1342.11) | 1248.62 (1163.88, 1339.52) |  |
