# Supplementary material for: Prediction of disability-adjusted life years for diseases due to low fruit intake in 2017–2040 in Japan
Source: Public Health Nutr. 2020 Nov 13;24(10):3156–66. doi: 10.1017/S1368980020004541 (PMC9884779; doi:10.1017/S1368980020004541)
Supplement: Supplementary file 1 [file S1368980020004541sup.zip › urn_cambridge.org_id_binary_20210107050822960-0641_S1368980020004541sup002.pdf]

Supplementary figure 1: Observed and projected 20–49 age group DALYs rate (per 100,000) for Neoplasms for reference and three alternative scenarios, 1990–2040: male, female and both sexes combined

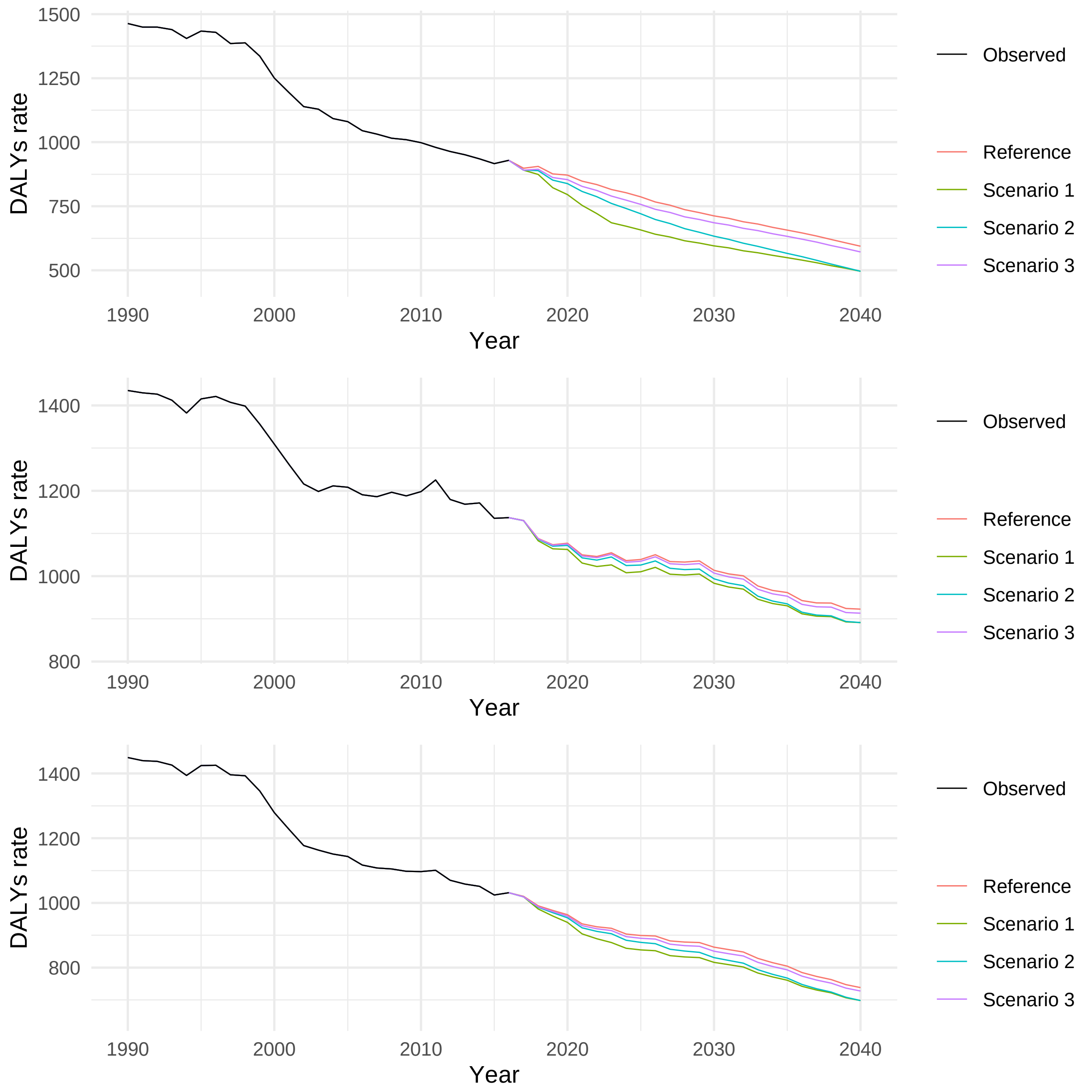

1: best scenario; 2: moderate scenario; 3: constant scenario

Supplementary figure 2: Observed and projected 50–69 age group DALYs rate (per 100,000) for Neoplasms for reference and three alternative scenarios, 1990–2040: male, female and both sexes combined

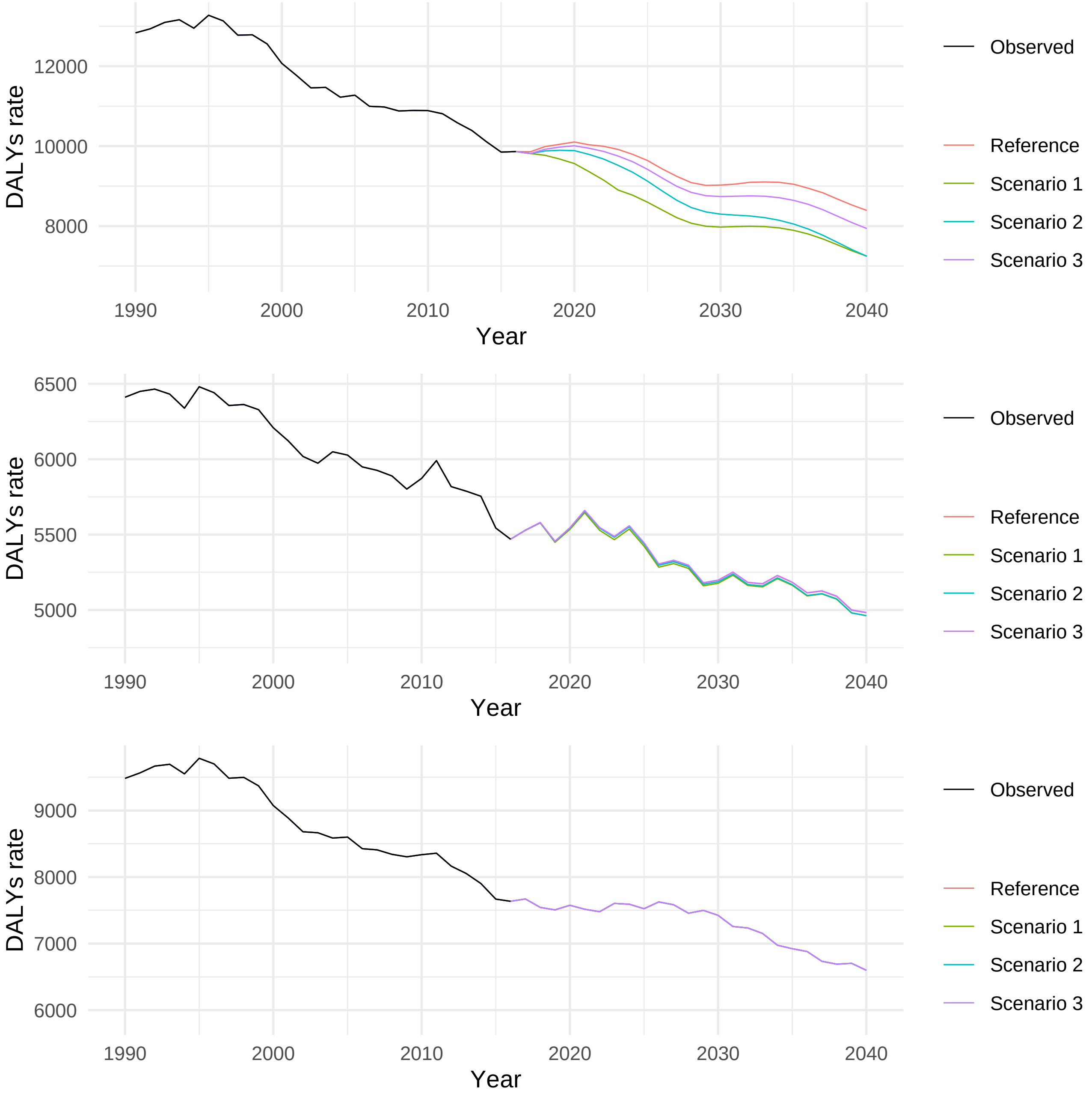

1: best scenario; 2: moderate scenario; 3: constant scenario

Supplementary figure 3: Observed and projected ≥70 age group DALYs rate (per 100,000) for Neoplasms for reference and three alternative scenarios, 1990–2040: male, female and both sexes combined

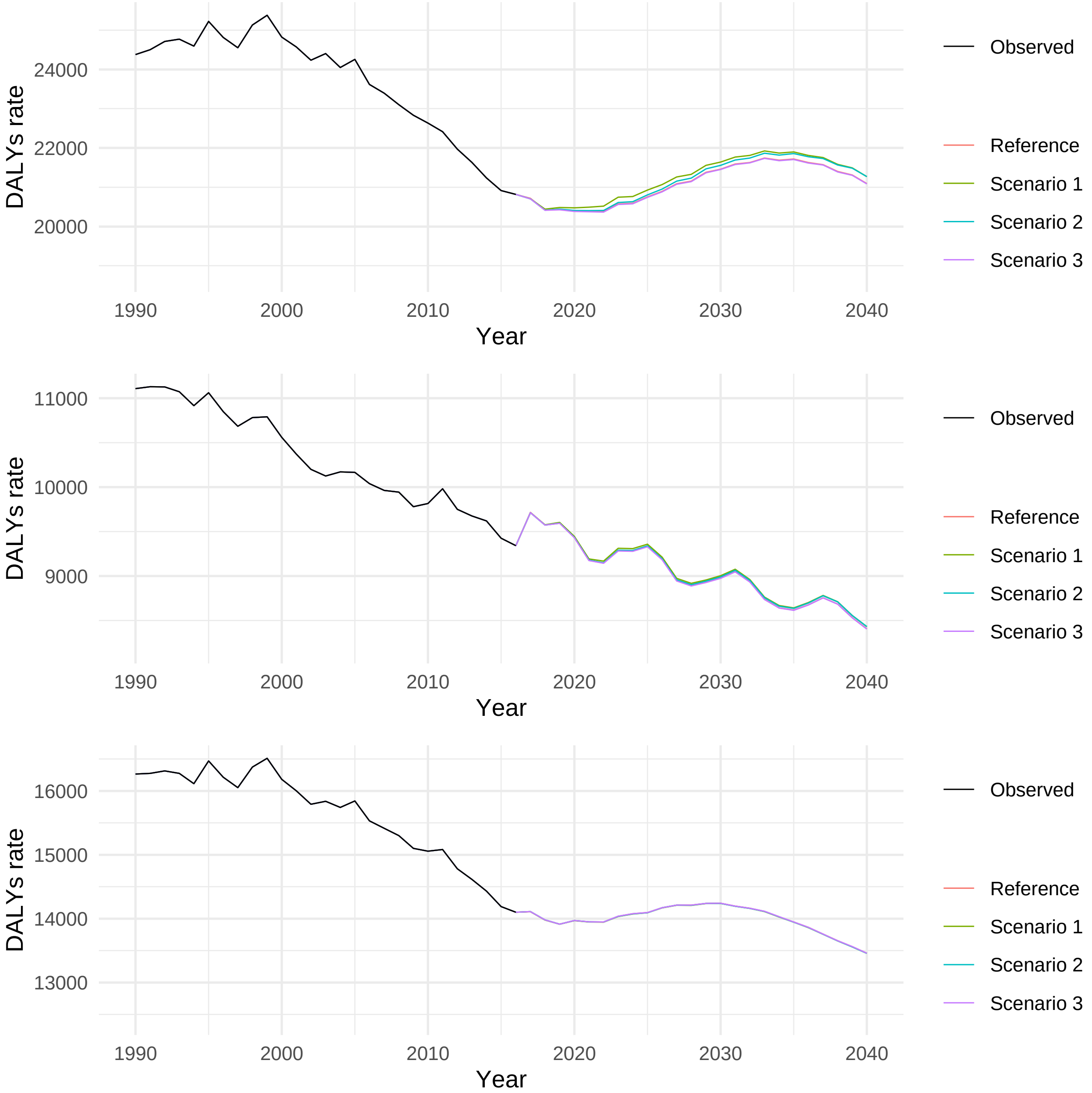

1: best scenario; 2: moderate scenario; 3: constant scenario

Supplementary figure 4: Observed and projected 20–49 age group DALYs rate (per 100,000) for cardiovascular diseases for reference and three alternative scenarios, 1990–2040: male, female and both sexes combined

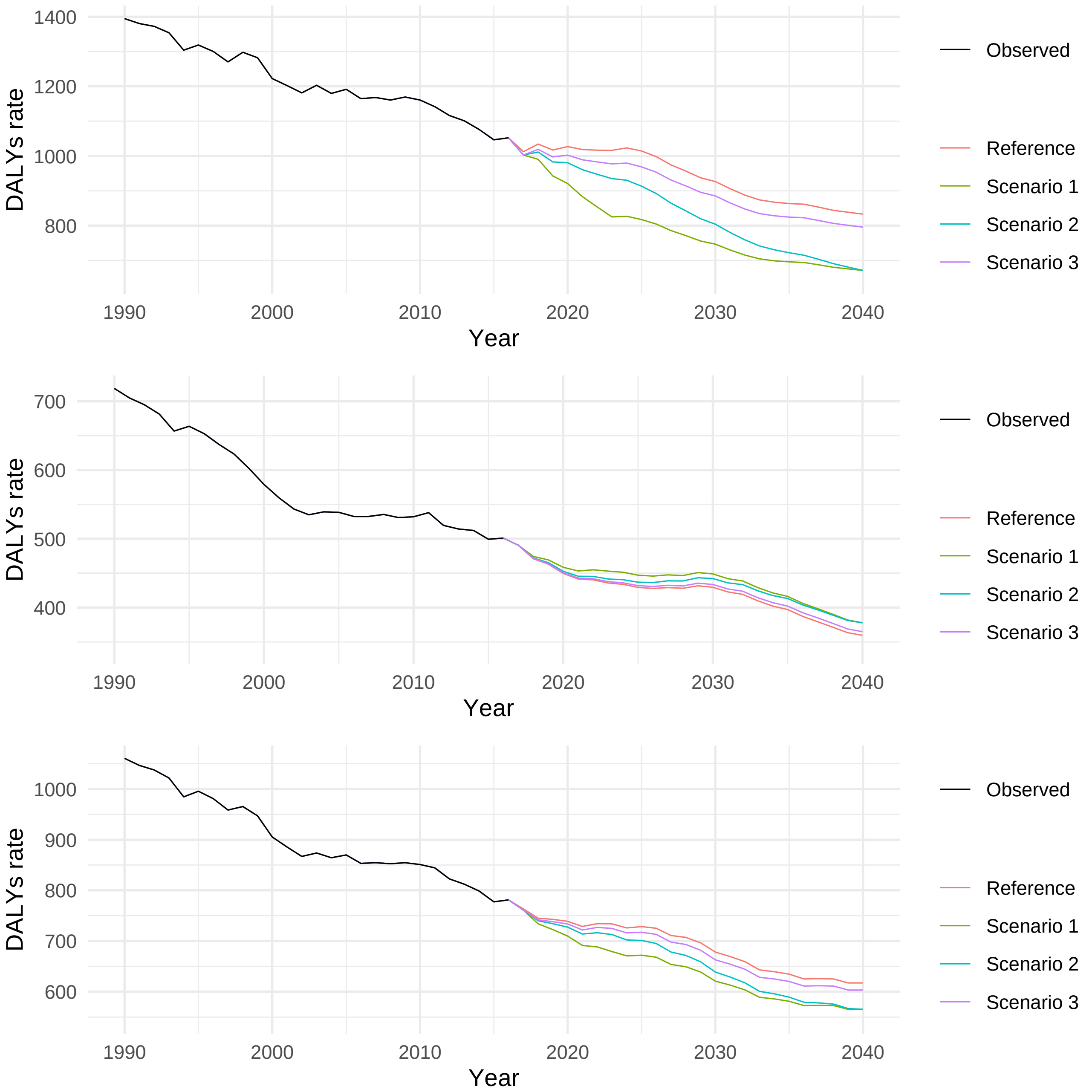

1: best scenario; 2: moderate scenario; 3: constant scenario

Supplementary figure 5: Observed and projected 50–69 age group DALYs rate (per 100,000) for cardiovascular diseases for reference and three alternative scenarios, 1990–2040: male, female and both sexes combined

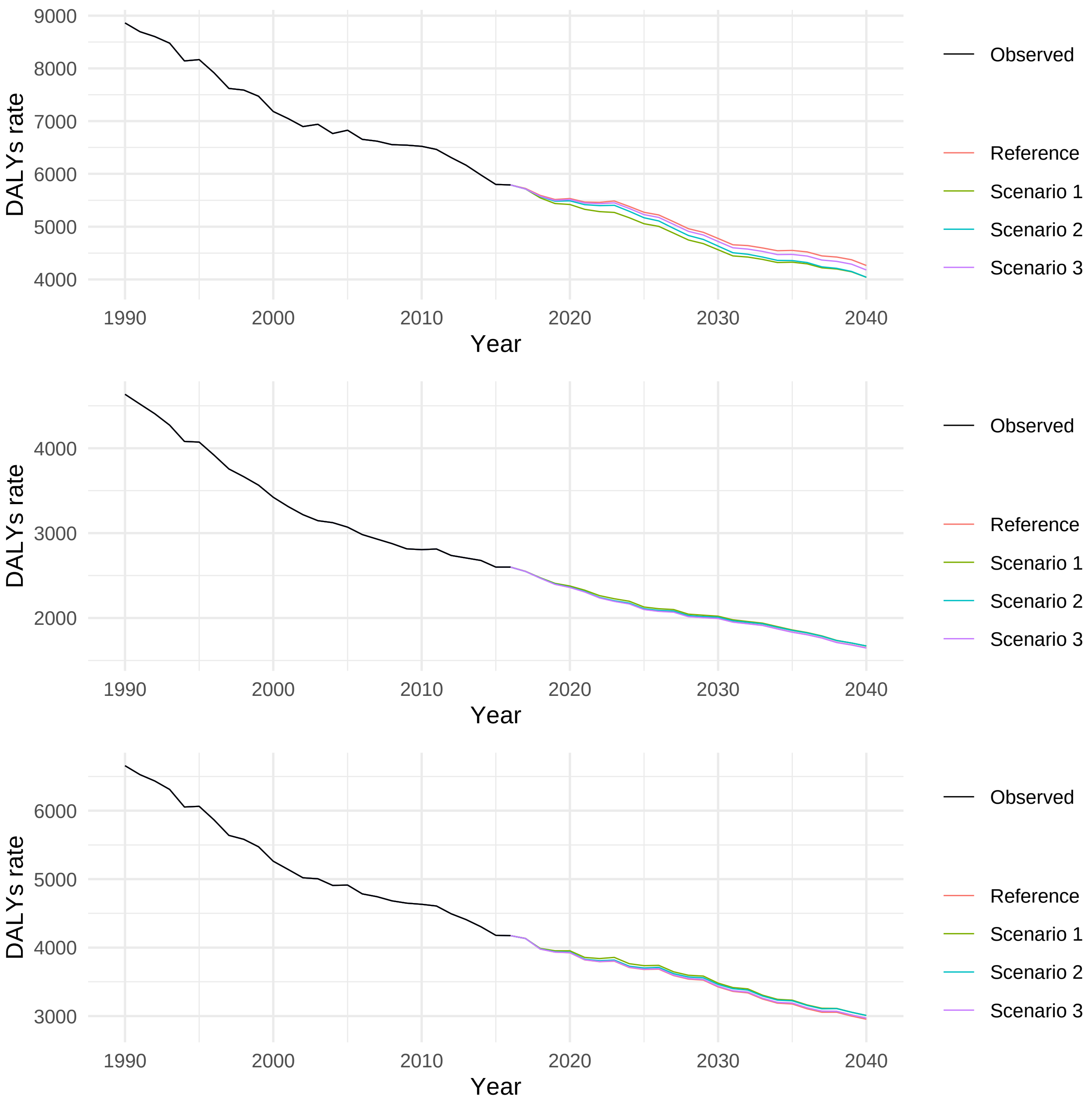

1: best scenario; 2: moderate scenario; 3: constant scenario

Supplementary figure 6: Observed and projected ≥70 age group DALYs rate (per 100,000) for cardiovascular diseases for reference and three alternative scenarios, 1990–2040: male, female and both sexes combined

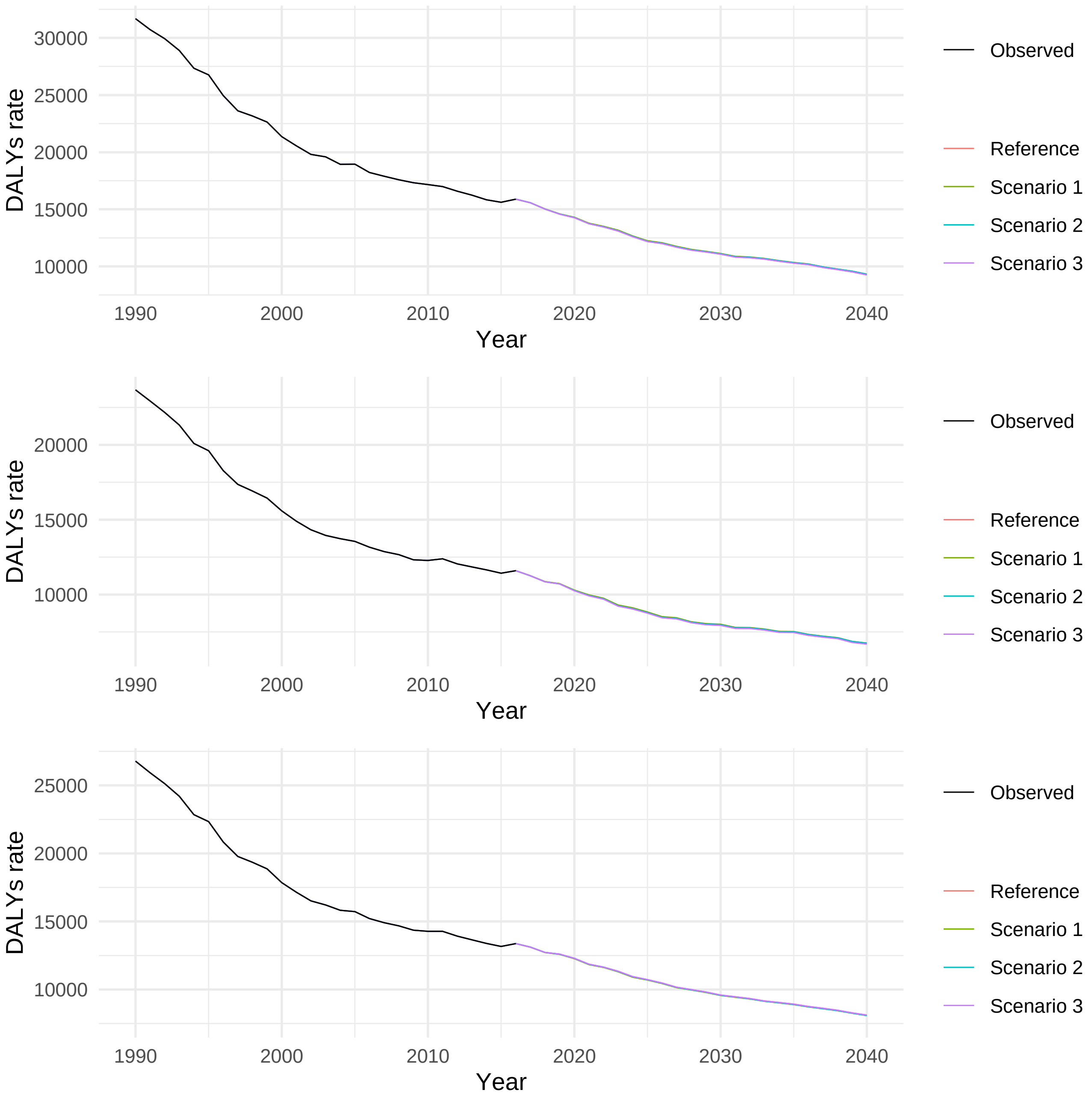

1: best scenario; 2: moderate scenario; 3: constant scenario

Supplementary figure 7: Observed and projected 20–49 age group DALYs rate (per 100,000) for diabetes and kidney disease for reference and three alternative scenarios, 1990–2040: male, female and both sexes combined

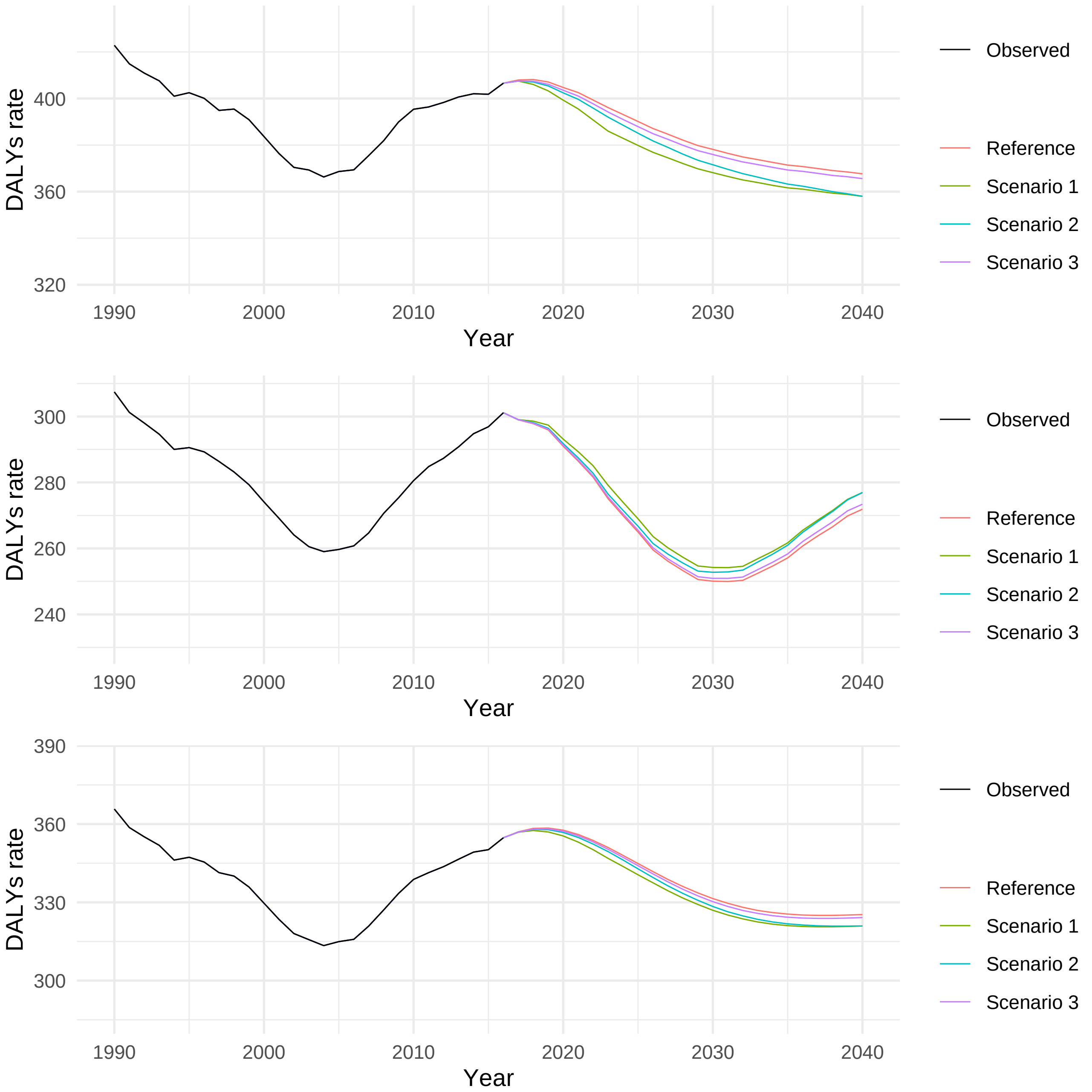

1: best scenario; 2: moderate scenario; 3: constant scenario

Supplementary figure 8: Observed and projected 50–69 age group DALYs rate (per 100,000) for diabetes and kidney disease for reference and three alternative scenarios, 1990–2040: male, female and both sexes combined

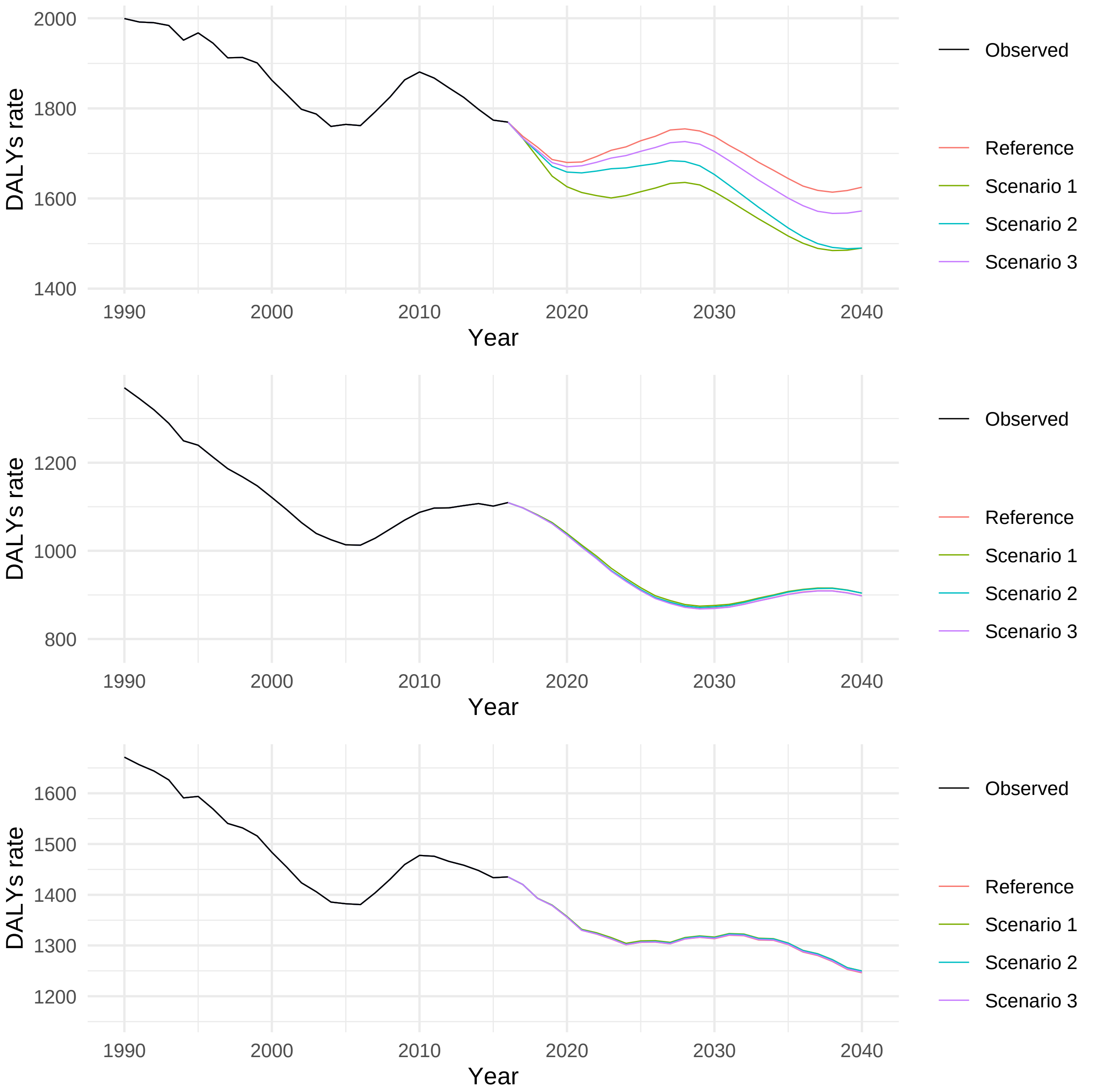

1: best scenario; 2: moderate scenario; 3: constant scenario

Supplementary figure 9: Observed and projected ≥70 age group DALYs rate (per 100,000) for diabetes and kidney disease for reference and three alternative scenarios, 1990–2040: male, female and both sexes combined

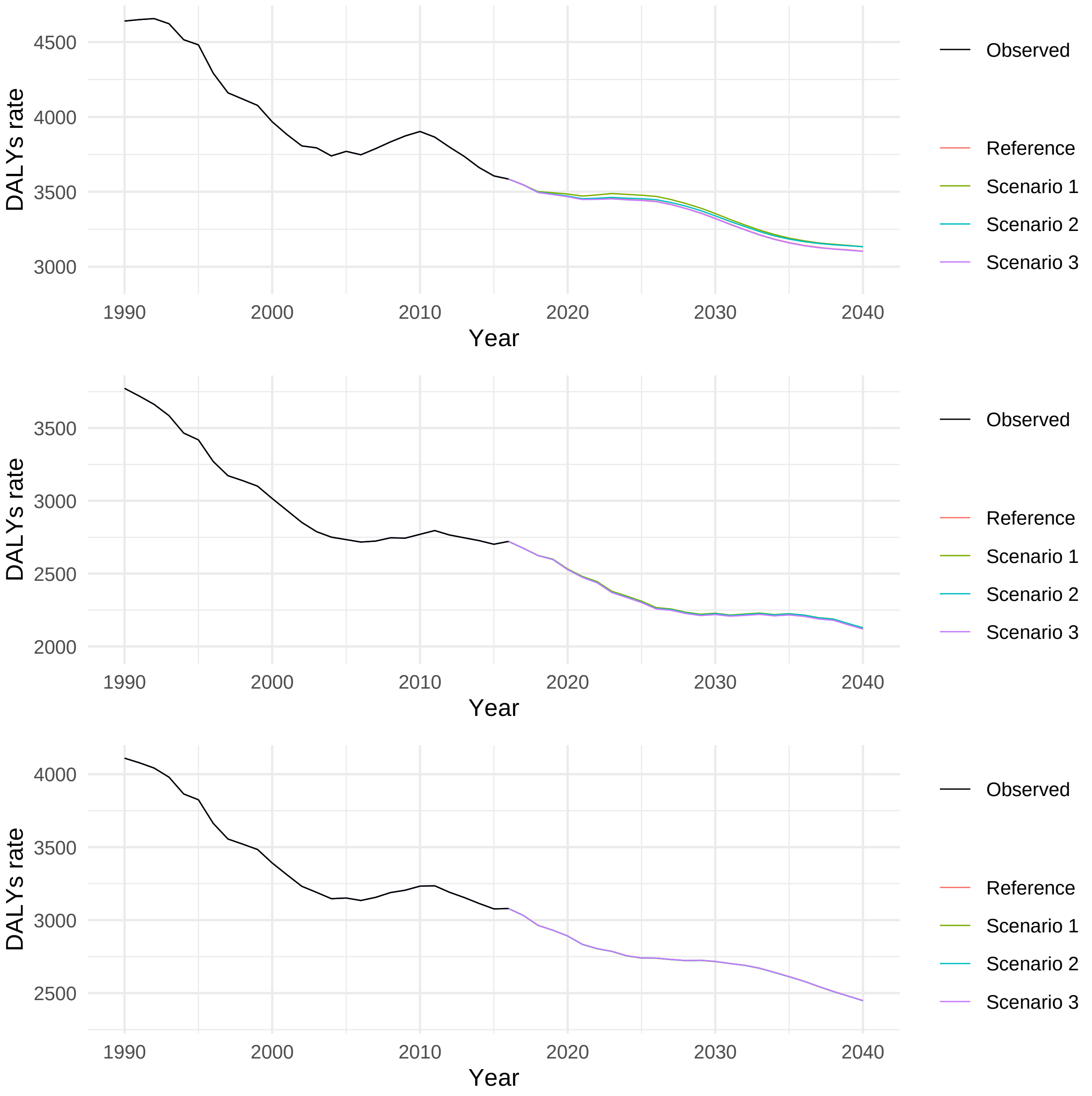

1: best scenario; 2: moderate scenario; 3: constant scenario
